# Multi-level analysis of the interactions between *REVOLUTA* and *MORE AXILLARY BRANCHES 2* in controlling plant development reveals parallel, independent and antagonistic functions

**DOI:** 10.1242/dev.183681

**Published:** 2020-05-21

**Authors:** Shin-Young Hong, Esther Botterweg-Paredes, Jasmin Doll, Tenai Eguen, Anko Blaakmeer, Sanne Matton, Yakun Xie, Bjørg Skjøth Lunding, Ulrike Zentgraf, Chunmei Guan, Yuling Jiao, Stephan Wenkel

**Affiliations:** 1 Copenhagen Plant Science Centre, University of Copenhagen, Thorvaldsensvej 40, 1871 Frederiksberg C, Denmark; 2 Department of Plant and Environmental Sciences, Faculty of Science, University of Copenhagen, Copenhagen, Denmark; 3 Centre for Plant Molecular Biology (ZMBP), University of Tübingen, Auf der Morgenstelle 32, 72076 Tübingen, Germany; 4 State Key Laboratory of Plant Genomics, Institute of Genetics and Developmental Biology, Chinese Academy of Sciences, and National Center for Plant Gene Research, Beijing 100101, China; 5 NovoCrops Center, PLEN, University of Copenhagen, Thorvaldsensvej 40, 1871 Frederiksberg C, Denmark

**Keywords:** Shade avoidance, Shoot branching, Senescence, Vascular development, HD-ZIPIII, *Arabidopsis*

## Abstract

Class III homeodomain leucine zipper (HD-ZIPIII) transcription factors play fundamental roles in controlling plant development. The known HD-ZIPIII target genes encode proteins involved in the production and dissipation of the auxin signal, HD-ZIPII transcription factors and components that feedback to regulate *HD-ZIPIII* expression or protein activity. Here, we have investigated the regulatory hierarchies of the control of *MORE AXILLARY BRANCHES2* (*MAX2*) by the HD-ZIPIII protein REVOLUTA (REV). We found that REV can interact with the promoter of *MAX2*. In agreement, *rev10D* gain-of-function mutants had increased levels of *MAX2* expression, while *rev* loss-of-function mutants showed lower levels of *MAX2* in some tissues. Like REV, MAX2 plays known roles in the control of plant architecture, photobiology and senescence, which prompted us to initiate a multi-level analysis of growth phenotypes of *hd-zipIII*, *max2* and respective higher order mutants thereof. Our data suggest a complex relationship of synergistic and antagonistic activities between REV and MAX2; these interactions appear to depend on the developmental context and do not all involve the direct regulation of *MAX2* by REV.

## Abstract (precis)

**Summary:** A complex relationship of synergistic and antagonistic activities exists between REV and MAX2 that appear to depend on the developmental context and do not all involve the direct regulation of *MAX2* by REV.

## INTRODUCTION

Plant development is highly plastic, and both leaf form and plant stature strongly depend on the environment a plant is exposed to. In suboptimal light conditions, e.g. in the canopy shade of other plants, *Arabidopsis* plants develop flattened leaves with extended petioles, show less branching and flower early. This suggests a tight connection between the pathways that control environmental responses and the gene networks that operate to control pattern formation in developing organs such as the leaves. In this context, the shoot apical meristem plays a decisive role as it maintains a pool of stem cells at the shoot tip. Cells that leave the stem cell niche at the lower side form stem tissue and cells at the flanks of the upper side produce new leaves. Factors controlling the establishment and maintenance of the shoot apical meristem have been identified decades ago. Members of the class III homeodomain leucine zipper (HD-ZIPIII) family of transcription factors play instrumental roles, starting with determining apical cell fate in the developing embryo (Smith and Long, 2010). Plants carrying loss-of-function mutations in three out of the five *HD-ZIPIII* genes, *REVOLUTA* (*REV*), *PHAVOLUTA* (*PHV*) and *PHABULOSA* (*PHB*), fail to establish a shoot meristem and develop pin-formed seedlings that arrest in development soon after germination (Emery et al., 2003; Prigge et al., 2005). *HD-ZIPIII* mRNA stability is controlled by the microRNA *miR165/6* (Emery et al., 2003). Gain-of-function mutations in *HD-ZIPIII* genes that disrupt microRNA regulation cause respective mRNAs to accumulate to high levels (Emery et al., 2003; McConnell et al., 2001) and mutant plants display strong developmental phenotypes. In the case of *PHB* and *PHV*, respective gain-of-function mutants often develop radialized leaves and have a stunted appearance (McConnell et al., 2001). The *rev10D* gain-of-function mutant displays radialized vasculature and leaf-to-stem fusions that cause bending of the main stem (Emery et al., 2003). Another miR-disrupting gain-of-function mutation in the *REV* gene is *amphivasal vascular bundle 1* (*avb1*), which causes similar growth defects as *rev10D* but additionally promotes the formation of ectopic axillary meristems (Zhong and Ye, 2004). Thus, *avb1* mutant plants have a very bushy appearance due to the outgrowth of side shoots from the axillary meristems. Interestingly, both *avb1* and *rev10D* carry the same mutation (P190 L) even though they have been isolated in different genetic screens. Thus, it appears that the differences in phenotype are either caused by variation in the growth conditions or by modifier mutations that segregate in the different genetic backgrounds. Loss-of-function mutations affecting *REV* (such as *rev5*) fail to initiate axillary meristems and have a barren appearance (Otsuga et al., 2001; Talbert et al., 1995), which agrees with REV being a positive regulator of shoot branching. Furthermore, REV directly activates the expression of *SHOOTMERISTEMLESS* to enable axillary meristem formation (Shi et al., 2016). Beside the negative regulation by microRNAs, HD-ZIPIII proteins are negatively controlled by LITTLE ZIPPER (ZPR)-type microProteins (Kim et al., 2008; Wenkel et al., 2007). MicroProteins are small single-domain proteins that are related in sequence to larger, multi-domain proteins and often act by establishing negative-feedback loops (Eguen et al., 2015; Staudt and Wenkel, 2011). *ZPR1*, *ZPR3* and *ZPR4* are direct and positive REV targets (Brandt et al., 2013). Thus, REV regulates its own protein activity by upregulating different ZPR microProteins.

Shoot branching is controlled by auxin in the course of establishing apical dominance of the main shoot. In this process, auxin is transported from the shoot to the root and represses the formation of side shoots (Booker et al., 2003). Strigolactones, plant hormones that are derivatives of the carotenoid pathway, have been identified as prominent branching suppressors by inhibiting outgrowth of lateral buds (Gomez-Roldan et al., 2008; Umehara et al., 2008). Recent work revealed that strigolactones and cytokinin function by affecting auxin transport (Shinohara et al., 2013; Waldie and Leyser, 2018) to control shoot branching. In this context, the *MORE AXILLARY BRANCHES* (*MAX*) genes encode components required for the production and perception of the strigolactone signal. Here, MAX1, MAX3 and MAX4 are biosynthetic enzymes that lead to the production of strigolactones (Booker et al., 2005; Stirnberg et al., 2002), while MAX2 is an F-Box protein that acts to initiate degradation of target substrates (Stirnberg et al., 2007). In *Arabidopsis*, it has been shown that MAX2 interacts with the strigolactone receptor D14 and upon hormone perception initiates the degradation of a SMXL6/7/8-type repressor complex (Wang et al., 2015).

*MAX2* was identified as a potential direct REV target gene (Brandt et al., 2012). Loss of *MAX2* function results in a pleiotropic phenotype and mutant plants have elongated hypocotyls, produce more side shoots and are delayed in leaf senescence. Here, we have validated that *MAX2* can be upregulated by REV and that both *REV* and *MAX2* have largely overlapping patterns of expression. To investigate the regulatory relationship between REV and MAX2 in the biological processes that they both seem to affect, we crossed available mutants to generate higher-order mutants. We assessed the phenotypic changes of respective single- and double-mutant plants that were grown with wild-type plants in different conditions. Our analysis revealed that shoot branching and hypocotyl elongation were subject to oppositional control by REV and MAX2. While REV promoted shoot branching and hypocotyl elongation, MAX2 acted as a suppressor in both processes. Hence, respective double mutants showed intermediate phenotypes. In the regulation of leaf senescence, however, both REV and MAX2 act as positive regulators and respective double mutants show partially additive phenotypes. During vascular development, REV acts as a polarizing factor that promotes the formation of xylem tissue (Zhong and Ye, 1999). Histological analysis revealed that the *rev10D*-induced radialization of vascular elements was suppressed in *rev10D max2* double mutant plants. This indicated that proteins that antagonize REV function might have accumulated in *max2* mutant plants. Taken together, this study uncovers context-dependent molecular mechanisms underlying REV functions in shoot and vascular architecture, photobiology, and senescence.

## RESULTS

### REVOLUTA is a positive regulator of *MAX2* expression

**Fig. 1.**
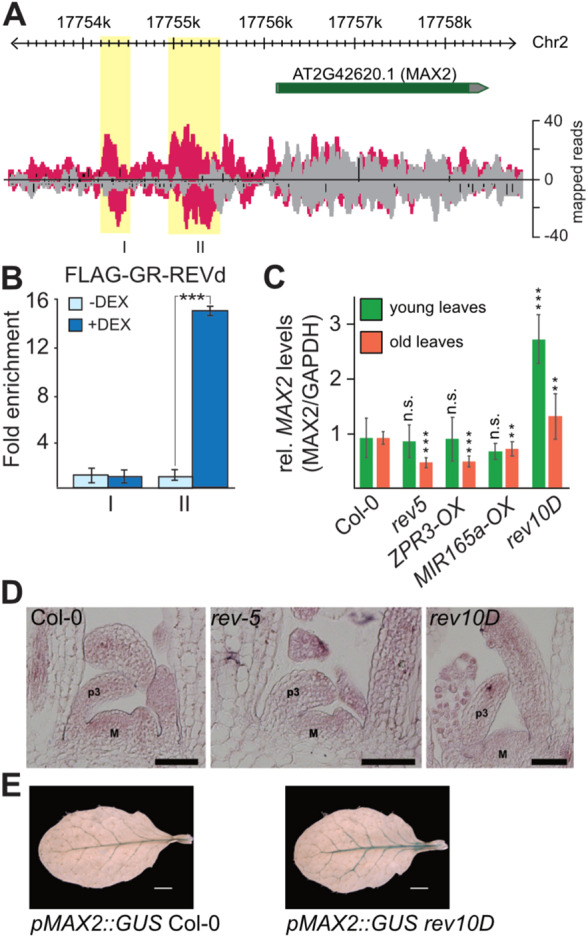
**REVOLUTA binds to the *MAX2* promoter and is a direct and positive regulator of *MAX2* expression.** (A) Read coverage at the *MAX2* locus obtained from ChIP-seq of Col-0 (gray) and DEX-induced *35S::FLAG-GR-REVd* plants (pink) (data from Brandt et al., 2012). The analysis reveals two potential binding sites (I and II) located −1.1 kb and −1.9 kb upstream of the transcription start site. (B) Chromatin-immunoprecipitation (ChIP-qPCR) experiments with three biological replicates for *35S::FLAG-GR-REVd* without DEX (light-blue bars) and *35S::FLAG-GR-REVd* with DEX (dark-blue bars) plants testing the two positions in the *MAX2* promoter. The *y*-axis shows the fold enrichment normalized to the non-induced IPs (-DEX). Data are mean±s.d. ***P<0.0005 (*t*-test). (C) Expression of *MAX2* was analyzed in different *rev* mutant plants (*rev5*, *rev10D*), in plants with genetic backgrounds of reduced *HD-ZIPIII* mRNA (*MIR165a-OX*) and in plants with reduced activity of HD-ZIPIII proteins (*ZPR3-OX*). Green bars indicate expression in young leaves (2-week-old plants); orange bars indicate expression in older leaves (4-week-old plants). Expression levels are calculated relative to the respective wild type, including the s.e.m. of three individual biological experiments **P<0.001, ***P<0.0005; n.s., not significant (Student's *t*-test). (D) *In situ* hybridization showing expression of *MAX2* in 12-day-old wild-type Col-0 (left), *rev5* (middle) and *rev10D* (right) plants. Indicated are leaf primordia (p3) and the meristem (M). (E) Analysis of the *pMAX2::GUS* reporter in Col-0 wild type (right) and *rev10D* in a rosette leaf. Scale bars: 50 μm in D; 2mm in E.

Expression of a dominant version of *REV* (*REVd*) fused to the rat glucocorticoid receptor under control of the *35S* promoter resulted in strong developmental defects when transgenic plants were additionally treated with dexamethasone (DEX) (Brandt et al., 2012). Here, DEX induces the translocation of the chimeric GR-REVd protein into the nucleus where it can interact with chromatin to control gene expression. This system was used to perform ChIP-seq studies and identify direct REV target genes (Brandt et al., 2012). The further study of REV targets revealed novel roles for REV in the shade avoidance response (Bou-Torrent et al., 2012; Brandt et al., 2014, 2012; Merelo et al., 2017, 2016) and the control of leaf senescence (Xie et al., 2014). Among the potential direct target genes, we also identified *MAX2*. The *max2* loss-of-function mutants are known to have an altered leaf morphology and increased branching (Stirnberg et al., 2002), delayed senescence (Woo et al., 2001) and a constitutive photomorphogenic response (Shen et al., 2007). Because several of these and related traits are also regulated by REV and the other HD-ZIPIII transcription factors in a redundant manner, we studied the relationship between REV and MAX2. With ChIP-seq we identified two potential REV binding sites in the *MAX2* promoter (Fig. 1A) located −1.1 and −1.9 kb upstream of the transcription start site. To validate binding of REV to either one or both of these sites, we performed independent ChIP-qPCR experiments. For this, we treated one set of our transgenic plants expressing FLAG-GR-REVd with DEX and the other set with a mock solution. No binding to the *MAX2* promoter was observed in the mock-treated samples. The analysis of the different binding sites revealed an interaction of REV with the more proximal region that was identified by ChIP-seq (Fig. 1B). Expression of *MAX2* was then analyzed in different *rev* mutant plants (*rev5*, *rev10D*), of which *rev5* plants are loss-of-function mutants and *rev10D* mutants are insensitive to miRNA regulation and resemble *REV*-overexpressing plants. Moreover, *MIR165a-OX* plants with reduced *HD-ZIPIII* mRNA (*MIR165a-OX*) and *ZPR3-OX* plants showing a reduced activity of HD-ZIPIII proteins, through an interaction with the overexpressed LITTLE ZIPPER3 protein, were also included (Fig. 1C). Analysis of young leaves revealed no changes in *MAX2* expression levels in genetic backgrounds with lost or reduced REV or HD-ZIPIII function (Fig. 1C). In the *rev10D* gain-of-function background, we detected a strong increase of *MAX2* mRNA, suggesting that HD-ZIPIIIs are not required to maintain the basal levels of *MAX2* expression at early stages of development but can act as positive regulators of *MAX2* expression. Analysis of older leaves revealed significantly reduced levels of *MAX2* expression in genetic backgrounds with lost or reduced REV or HD-ZIPIII function and slightly increased levels in *rev10D* mutant plants. Furthermore, the analysis of *MAX2* expression in different tissues showed a reduced expression of *MAX2* in flowers of plants overexpressing *ZPR3* and *MIR165a* (Fig. S1A,B). These findings support a role for REV as a positive regulator of *MAX2* expression, especially at later stages of development.

We also assessed the spatial pattern of *MAX2* expression in *rev5* and *rev10D* mutant plants by *in situ* hybridization. We observed that *MAX2* expression was slightly lower in *rev5* mutant plants compared with the Col-0 wild type. In *rev10d* mutant plants, we observed signal intensities that were comparable with the Col-0 wild type (Fig. 1D). Analysis of the *pMAX2::GUS* reporter in wild-type and *rev10D* mutant plants yielded stronger GUS expression in the latter mutant background (Fig. 1E). Moreover, the GUS signal was, in both cases, restricted to the vasculature, indicating that REV is not active outside its expression domain. In summary, these findings support a role for REV as an upstream regulator of *MAX2* in adult tissue that can be modulated in response to age or physiological status.

### REV and MAX2 have largely overlapping patterns of expression

**Fig. 2.**
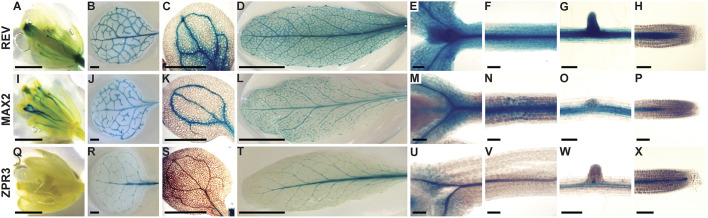
**Spatial expression analysis of *REV*, *MAX2* and *ZPR3*.** Patterns of expression of *REV* (A-H), *MAX2* (I-P) and *ZPR3* (Q-X) in 10-day-old *Arabidopsis* seedlings, and 4-week- and 8-week-old *Arabidopsis* tissues. (A,I,Q) Expression of *promoter::GUS* fusions in flower. Scale bars: 1 mm. (B,J,R) Young rosette leaves. Scale bars: 1 mm. (C,K,S) Cotyledon. Scale bars: 1 mm. (D,L,T) Developing rosette leaves. Scale bars: 1 cm. (E,M,U) Shoot apex. Scale bars: 0.1 mm. (F,N,V) Hypocotyl. Scale bars: 0.1 mm. (G,O,W) Root with root hair. Scale bars: 0.1 mm. (H,P,X) Root tip. Scale bars: 0.1 mm.

REV can upregulate *MAX2* expression in a direct manner. We next compared the spatial patterns of expression of *REV*, *LITTLE ZIPPER3* (*ZPR3*; a known direct REV target gene) and *MAX2*. To this end, we analyzed respective promoters fused to the β-*glucuronidase* (*GUS*) gene in stably transformed plants. We reasoned that if REV acts on *MAX2*, we should observe largely overlapping patterns of expression of all three genes. In flowers, strong GUS staining indicative of *REV* expression was detected at the base and top of the pistil and the stamens (Fig. 2A), whereas *MAX2* was expressed only in stamens (Fig. 2I) and *ZPR3* expression was not detectable (Fig. 2Q). In cotyledons, and young and old rosette leaves, *REV* and *MAX2* expression was detectable in the vasculature, while *ZPR3* expression was also vascular localized but very weak in the vasculature of cotyledons (Fig. 2B-D,J-L,R-T). At the shoot apex, strong GUS signal indicative of *REV* and *MAX2* expression was observed in the shoot meristem region (Fig. 2E,M), while *ZPR3* expression was restricted to the vascular strands that frame the meristem (Fig. 2U). In hypocotyls and roots, we observed largely overlapping patterns of expression of all three genes and here the GUS signals were mostly present in the vasculature (Fig. 2F-H,N-P,V-X). Taken together, we found that all genes are expressed at different levels and they exhibited partially overlapping patterns of expression, indicative of a regulatory relationship. This suggests that REV has the potential to act as a modulator of *MAX2* expression.

### REV controls shoot branching in *Arabidopsis*

**Fig. 3.**
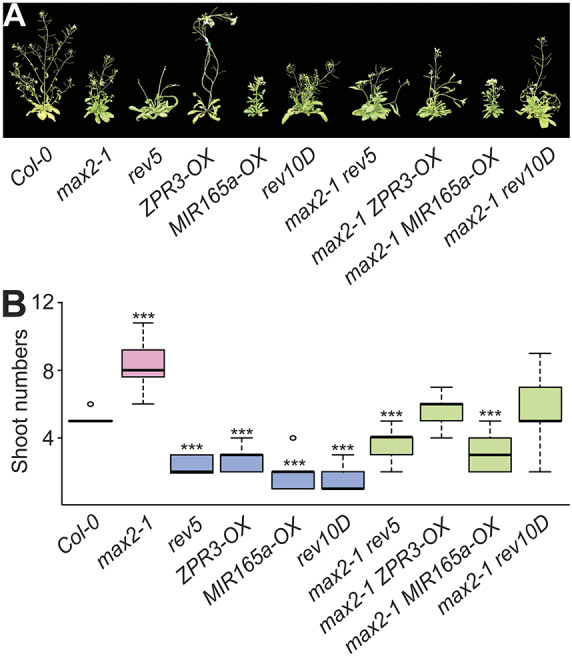
***REV* and *MAX2* regulate shoot branching.** (A) Representative images of 6-week-old plants grown under long-day conditions. (B) Quantification of the total number of shoots produced in Col-0 (wild type, white), single mutant plants (pink and blue) and double mutant plants (green). A two-tailed *t*-test was used to test the significance relative to the Col-0 wild type. ***P<0.0005. *n*=7-15.

MAX2 has a known function as a branching inhibitor in *Arabidopsis*; in *max2* loss-of-function mutants axillary shoots grow out and plants have a bushy appearance. In contrast, REV plays a known role as activator of shoot branching and *rev* loss-of-function mutants develop fewer side shoots. To investigate the genetic relationship of *REV* and *MAX2* with regard to their role in shoot branching, we generated sets of double mutants with *max2* in combination with *rev* gain- and loss-of-function mutants. As observed before, we detected a higher number of side shoots in *max2* mutant plants compared with the corresponding Col-0 wild type (Fig. 3A,B, Table 1). In line with the known functions of REV, we observed reduced shoot numbers in *rev5* mutant plants, as well as in transgenic plants overexpressing either *miR165a* (*MIR165a-OX*) or the *ZPR3* microProtein (*ZPR3-OX*), which leads to a reduction in *HD-ZIPIII* RNA levels or in HD-ZIPIII activity, respectively. In contrast to our expectation, we also observed reduced numbers of side shoots in the *rev10D* gain-of-function mutant (Fig. 3A,B). However, a closer inspection of this mutant revealed that the main stem appears fasciated and is likely a product of a fasciated meristem producing an unknown number of shoot fusions precluding detailed analysis. The combination of *max2* with the different *REV/HD-ZIPIII* loss-of-function mutants revealed a strong suppression of the *max2* phenotype and double mutant plants resembled the *REV/HD-ZIPIII* loss-of-function mutants (Fig. 3A,B, Table 1). The finding that the *max2* phenotype was suppressed by *rev* loss-of-function mutations implies that the *max2* mutant phenotype is dependent on the activity of REV. However, as the double mutants were not completely identical to the *REV/HD-ZIPIII* loss-of-function mutants, we conclude that REV and MAX2 play at least partially independent roles in the regulation of shoot branching.

**Table 1.**
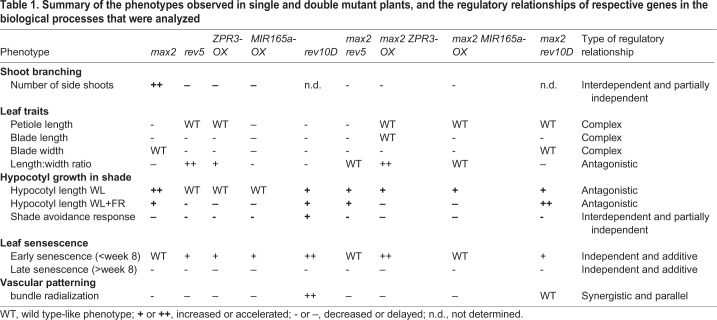
**Summary of the phenotypes observed in single and double mutant plants, and the regulatory relationships of respective genes in the biological processes that were analyzed**

### *MAX2* and *HD-ZIPIII* genes influence different aspects of leaf morphology

Plants carrying loss-of-function mutations in *MAX2* not only show branching defects but have also altered rosette and leaf morphologies. Compared with wild type, *max2* mutants develop a more compact rosette with rounder leaves, which strongly resembles that in the *rev10D* mutants (Fig. S2A). Measuring the lengths of the petioles of wild type and the different single and double mutant plants revealed that all single mutants tend to have shorter petioles compared with the wild type. The *rev5* and *ZPR3-OX* plants have petioles comparable with Col-0 wild-type plants, while *MIR165a-OX* plants have strongly reduced petiole lengths. The latter indicates that ectopic microRNA overexpression produces more-complex phenotypes compared with the loss of *REV* function or the *ZPR3* overexpression. It is well established that *miR165/6* interacts with both ARGONAUTE1 (AGO1) and the PINHEAD/ZWILLE/AGO10: here, AGO1 initiates miR-dependent *HD-ZIPIII* degradation (Kidner and Martienssen, 2004), while AGO10 sequesters *miR165/6*, leading to stabilization of *HD-ZIPIII* mRNAs (Zhu et al., 2011). The *rev10D* and *max2* single mutants both have significantly reduced but similar petiole lengths (Fig. S2B). This contrasts with *max2 rev10D* double mutants, which have petiole lengths comparable with wild type, suggesting a complex interaction. In mutant plants with rounder leaves (such as *rev10D* and *max2*), quantification was easier compared with mutant plants that had narrower, downward curling leaf blades (such as *rev5*, *ZPR3-OX* and *MIR165a-OX*). In the latter mutants it was sometimes difficult to pinpoint the position at which the blade ended and the petiole started. The analysis of respective double-mutant plants revealed petiole length similar to the Col-0 wild type, except *max2 rev5* that had slightly shorter petioles, which is again indicative of a complex genetic interaction. In addition to measuring petiole length, we also assessed the overall leaf morphologies by measuring both leaf lengths and leaf widths. As pointed out before, *max2* mutant plants had rounder leaves with a length-to-width ratio of around 1.3, compared with Col-0 wild-type leaves that were more elongated with a length-to-width ratio of around 1.7 and *rev10D* with a ratio around 1.5 (Fig. S2C). Transgenic *ZPR3-OX* plants and *rev5* mutant plants had downward curled narrow leaves with length-to-width ratios of around 2.1 and 2.4, respectively. Transgenic *MIR165a-OX* plants had a low length-to-width ratio (around 1.5) but were severely stunted. The analysis of respective double-mutant plants revealed wild-type-like leaf morphologies for *max2 rev5* and *max2 MIR165A-OX* double mutants, with a length-to-width ration of around 1.6 (Fig. S2A,C), while the leaf morphologies of transgenic *max2 ZPR3-OX* plants resembled the respective transgenic lines with wild-type *MAX2* alleles. Taken together, we can conclude that REV and MAX2 play opposing roles in regulating leaf morphology: REV acts as a positive regulator, promoting leaf width; MAX2 acts as a negative regulator, restricting leaf width. The combination of both mutants generates an intermediate phenotype and respective double mutants resemble the Col-0 wild type. This indicates that, under these circumstances, the direct regulation of *MAX2* through REV appears to play a subordinate role and other factors appear to be more important.

### REV enables distinct MAX2-dependent responses in the promotion of hypocotyl growth under the shade

**Fig. 4.**
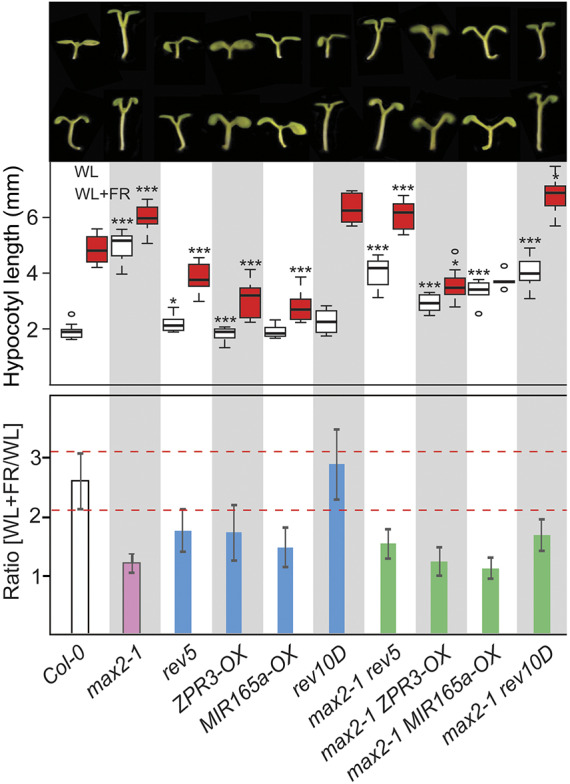
**REV and MAX2 have independent and antagonistic roles in the promotion of hypocotyl growth in response to shade.** Seeds of respective wild-type and mutant plants were cultivated for 2 days in white light (WL) conditions and subsequently cultivated for 7 days in either WL or far-red-enriched white light (WL+FR) conditions. The upper panel depicts representative seedlings. Box plots in the middle panel show the quantification of hypocotyl lengths for the various genotypes; white boxes, hypocotyls grown in WL; red boxes, hypocotyl grown in WL+FR. *P<0.05, ***P<0.0005, Student's *t*-test relative to the hypocotyl lengths of wild type. *n*=20-25. Plots show median values (middle bars) and 25th and 75th percentiles (boxes); whiskers indicate 1.5× the interquartile ranges. The lowest panel depicts the relative hypocotyl extension in shade by dividing the lengths of the hypocotyl in WL+FR with the lengths observed in WL conditions. Dashed lines (red) indicate the range of relative extension observed in the Col-0 wild type.

*Arabidopsis* is a shade-sensitive plant that reacts to both qualitative and quantitative changes of light. When shaded by other plants, the ratio of the red to far-red light is reduced and plants show a set of growth responses that include hypocotyl elongation, petiole elongation, premature flowering and early senescence, commonly referred to as shade-avoidance syndrome (Roig-Villanova and Martinez-Garcia, 2016). To determine how wild-type and mutant plants respond to shade, we grew seedlings in white light growth chambers equipped with additional red and far-red LEDs. This setup allowed us to keep the photosynthetic active radiation (PAR) at a constant level but specifically alter the red to far-red light ratio. When grown in white light (WL), Col-0 wild-type plants showed very short hypocotyls compared with seedlings grown in shade (WL+FR), which developed very long hypocotyls (Fig. 4). In both WL and WL+FR, *max2* mutant plants had very long hypocotyls and elongation in shade was strongly reduced, resulting in a growth ratio (hypocotyl length in WL+FR/hypocotyl length in WL) close to 1. Plants carrying mutations in REV (here *rev5*) or transgenic plants with reduced HD-ZIPIII mRNA or HD-ZIPIII activity, as in *MIR165a-OX* and *ZPR3-OX*, respectively, showed normal hypocotyl length when grown in WL but displayed much shorter hypocotyls when grown in WL+FR. This is consistent with previous findings (Brandt et al., 2012). In addition, *rev5* mutants, and *MIR165a-OX* and *ZPR3-OX* transgenic plants had growth ratios smaller than wild-type plants, which is indicative of a disturbed shade response, supporting a positive role for HD-ZIPIIIs in the shade-avoidance response. In agreement, *rev10D* mutant plants appeared mildly hypersensitive to shade and developed slightly longer hypocotyls in both WL and WL+FR conditions, with an increased growth ratio (Fig. 4). The *max2 rev5* double-mutant plants had slightly shorter hypocotyls in WL compared with *max2* single mutants, indicating the constitutive growth in WL requires REV. When assessing the shade-avoidance response, it seems that respective double mutant regained the ability to respond to shade with a growth ratio comparable to that of *rev5*. The combination of *max2* with either *miR165a-OX* or *ZPR3-OX* resulted in a complete shade insensitivity, with shorter hypocotyls in both WL and WL+FR conditions (relative to the *max2* single mutant); *max2 rev10D* double mutants developed long hypocotyls in WL but were able to significantly elongate in WL+FR conditions, indicating that enhanced REV expression can partially rescue the shade-insensitive phenotype of *max2* (Fig. 4). In summary, these results confirm a positive role for REV in promoting elongation growth in shade and a negative role for MAX2 as a suppressor of elongation growth.

### MAX2 and REV are involved in leaf senescence progression

**Fig. 5.**
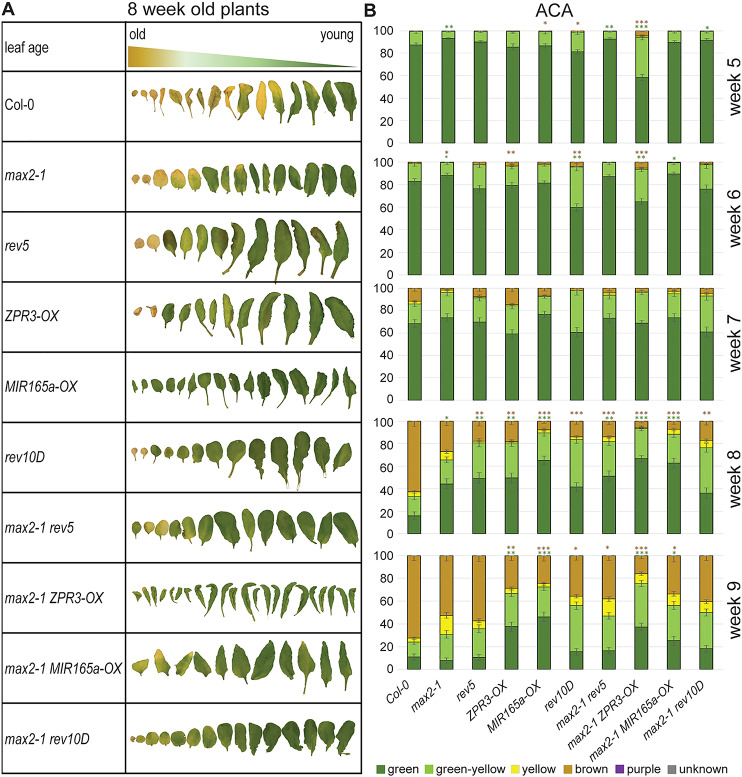
**Colorimetric analysis shows delayed senescence for mutant lines compared with wild type.** (A) Representative pictures of rosette leaves of 8-week-old plants, sorted according to their age. (B) Automatic color detection of rosette leaves by ACA. Six color categories were defined (fully green, green-yellow, fully yellow, brown or dry, and purple, with unknown color indicated in gray). The percentages of each color group are assigned for wild-type and mutant lines in weeks 5-9. Data are mean±s.e.m, *n*=6. Kruskal–Wallis test was performed for calculating statistically significant differences of all values at each timepoint compared with Col-0 (*P≤0.05, **P≤0.01, ***P≤0.001).

Regulation of leaf senescence is another trait influenced by REV. Overlapping patterns of expression of *MAX2* and *REV* were observed in older leaves and therefore we examined the involvement of *MAX2* and *HD-ZIPIII* mutants during the onset and progression of senescence. We analyzed different senescence-related parameters to gain a comprehensive understanding. Leaves of one rosette were sorted according to their age and photographed, and leaves of the same position in the rosette were compared. One typical example of 8-week-old plants is presented in Fig. 5A, already indicating differences in senescence strength. In order to quantify these differences, leaves of the same position of at least six plants were harvested in a weekly rhythm from week 5 to week 9. Visible phenotypical changes in leaf color due to chlorophyll degradation were categorized using an automated colorimetric assay (ACA; Bresson et al., 2018) in which the leaf color is analyzed pixel-wise and the percentage of a specific color is calculated relative to the whole leaf area (Fig. 5B). Compared with wild type (Col-0), *rev5* mutant plants showed a delay in senescence progression, while overall development was not retarded. Likewise, *max2* mutant plants were delayed in senescence progression; however, the delay appears to be more pronounced at the beginning of senescence in 5- and 6-week-old plants, whereas at later stages *rev5* plants appear to be significantly delayed, indicating that REV regulates additional senescence-associated genes. The *rev5 max2* double mutant displays an additive effect: while it resembles more the *max2* phenotype in 5- and 6-week-old plants, it is very similar to the *rev5* phenotype in 8-week-old plants and more senescent than both single mutants in 9-week-old plants. These findings support a role for REV and MAX2 in the same physiological pathway in which they are both main players, but additional independent effects also exist. Plants with reduced *HD-ZIPIII* mRNA levels by *microRNA165a* overexpression (*MIR165a-OX*) and plants with reduced activity of HD-ZIPIII proteins by interaction with LITTLE ZIPPER3 proteins (*ZPR3-OX*) revealed that not only REV but also other HD-ZIPIII proteins are involved in senescence regulation, as their phenotype is even more severely delayed in senescence progression than *rev5.* This is especially visible in later stages of development. When the *max2* mutation was introgressed into the genetic background of reduced *HD-ZIPIII* mRNA (*MIR165a-OX*), plants displayed a very similar phenotype to *MIR165a-OX* plants, suggesting that HD-ZIPIII proteins also have target genes besides *MAX2*. Interestingly, an additional effect was observed when HD ZIPIII protein activity, but not mRNA levels, was inhibited by the overexpression of *ZPR3*. When the *max2* mutant was combined with reduced activity of HD-ZIPIII proteins (*ZPR3-OX*), this line showed an enhanced senescence phenotype in 5- and 6-week-old plants compared with *max2* or *ZPR3*-overexpressing plants, but progression was almost completely arrested in the two following weeks so that in 8-week-old plants senescence was similarly delayed compared with *max2 MIR165a-OX*. As the MAX2 protein is absent due to mutation of the gene in the *max2 ZPR3-OX* plants, this could indicate that a potential MAX2 protein interaction partner might be liberated in early stages of development (5 and 6 weeks) and could inhibit ZPR3 action. As the *max2 ZPR3-OX* line showed first accelerated and then delayed senescence compared with *rev5 max2* double mutants, the interplay between MAX2, HD ZIPIII and LITTLE ZIPPER proteins appears to be more complex and differs in early and late stages. In addition, the miRNA-insensitive *rev10D* mutant shows an antagonistic phenotype to the loss-of-function allele, but only at the beginning of senescence in 5- and 6-week-old plants. At later time points, *rev10D* mutant and *max2 rev10D* double mutant plants also exhibit delayed senescence, again pointing at a more complex regulatory network between MAX2, HD-ZIPIIIs, LITTLE ZIPPER proteins and miRNAs during senescence. Besides the phenotypic observations, the analyses of further physiological senescence-related parameters confirmed these results. The pulse amplitude modulation (PAM) method images the chlorophyll fluorescence and describes the activity of photosystem II. This activity declined earlier in the Col-0 leaves compared with all mutants. This decline was more pronounced in *max2* than in *rev5* plants, and the *max2 rev5* double mutants showed an intermediate course (Fig. S3A), which is consistent with the phenotypic analyses. In addition, in all *max2* mutants with other backgrounds, photosystem II activity also remained higher compared with the *max2* in Col-0 background, which also matched the phenotypic analyses. In later stages of senescence, plasma membranes become more and more fragile and permeable, finally leading to the collapse of the cells. The membrane integrity was measured by ion leakage (Fig. S3B) and further supports the results of the colorimetric analysis. Col-0 displayed higher membrane deterioration than all other mutants and *max2* plants were more similar to Col-0 than to *rev5* plants and respective double mutants. Consistent with the accelerated senescence phenotype, *max2 ZPR3-OX* had a slightly higher membrane deterioration than wild-type plants in 6-week-old leaves. However, *rev10D* did not show the same effect, even though it also showed a very similar accelerated senescence phenotype to *max2 ZPR3-OX*, again indicating that the interplay appears not to be simple. The same held true for intracellular hydrogen peroxide contents. Hydrogen peroxide serves as a signal molecule for the initiation and progression of leaf senescence. Therefore, we measured the intracellular ROS contents by H_2_DCFDA fluorescence (Fig. S3C). All mutant lines had a lower H_2_O_2_ content compared with wild type in later stages of senescence, which is consistent with the delayed progression of senescence of all tested plant lines. Here, *max2 rev10D* levels appear to be very similar to the wild type in 8-week-old plants, even though senescence was delayed in this line, suggesting that different parts of the senescence program are altered in the different mutants.

Finally, we analyzed the expression of leaf senescence marker genes by quantitative real-time PCR. Therefore, we chose three senescence-associated genes (SAGs) *SAG12* and *SAG13*, encoding a cysteine protease and a short-chain alcohol dehydrogenase, respectively, as well as a NAC-domain transcription factor (ANAC092/ORE1). *SAG12* is often used as a marker gene in late senescence and therefore is not detectable in week 6. In week 7, Col-0 plants reached the highest expression level, whereas low levels could be detected in *max2*, *rev5* and *ZPR3-OX.* For the other mutant lines, *SAG12* expression starts even later, suggesting a more delayed senescence process (Fig. S3D). The expression levels of *SAG13* (Fig. S3E) were also highest in wild-type plants, but the differences in expression to the mutants were not very pronounced, which is consistent with the role of *SAG13* in early senescence and the delay of the senescence progression in the mutants at later stages. For *ANAC092* (Fig. S3F) a similar pattern to that of *SAG13* was observed, but in this case the expression in all 8-week-old mutant plants was clearly diminished compared with wild type. Interestingly, *ANAC092* expression was slightly higher in 6-week-old *ZPR3-OX* and *rev10D* plants, which corresponded to the accelerated phenotype of these two lines in this early stage.

Taken together, we can conclude that MAX2 and REV, as well as some other HD-ZIPIIIs, act in the same pathway to control the progression of senescence; however, MAX2 and REV also have independent effects. Overall, a complex regulatory network between HD-ZIPIII, miRNAs, LITTLE ZIPPER proteins and the different target genes appears to be at work.

### REV regulates additional genes involved in shoot branching and growth control

To further explore the independent functions of REV in the regulation of shoot branching, we investigated potential direct REV target genes that operate at the nexus of growth regulation. We performed comparative analysis of available ChIP-seq, RNA-seq and microarray data (Brandt et al., 2012; Reinhart et al., 2013), and paid particular attention to genes with known roles in hormonal signaling pathways. Brassinosteroid hormones act as global growth regulators. Mutations in genes encoding enzymes that synthesize the brassinosteroid hormone or receptors that perceive the hormone show a severe dwarf phenotype. *BAS1* encodes an enzyme that acts in the brassinosteroid catabolism and *bas1-D* gain-of-function mutants suppress hypocotyl growth (Neff et al., 1999). In organ boundaries, LATERAL ORGAN BOUNDARIES (LOB) suppresses brassinosteroid actions by inducing *BAS1* (Bell et al., 2012). Previous ChIP-seq and microarray data show that REV can upregulate *BAS1* expression. We performed gene expression analysis of *BAS1* with transgenic *35S::FLAG-GR-REVd* plants treated with either a mock solution or a dexamethasone-solution (DEX) that induces the translocation of the FLAG-GR-REVd protein from the cytoplasm to the nucleus. In addition, plants were pre-treated with cycloheximide, a protein biosynthesis inhibitor, allowing us to identify only direct REVd target genes. In support of the previous findings, we found that REVd induced *BAS1* expression (Fig. S4).

The hormone abscisic acid (ABA) acts as a stress hormone and controls seed germination but has recently been shown to inhibit axillary bud outgrowth. ABA is induced by a transcriptional cascade in developing shoot buds to suppress branching under suboptimal light conditions (González-Grand; et al., 2017). Several of the potential REV target genes play a role in ABA-related signaling. The GTL1 transcription factors control drought tolerance (Yoo et al., 2010), HSL1 and PIF1 control seed germination (Oh et al., 2004; Zhou et al., 2013), and SnRK3.9 is a protein kinase active in polarized pollen tube growth (Steinhorst et al., 2015); however, several related kinases act in salt signaling (Barajas-Lopez et al., 2018). We found that *GTL1*, *HSL1*, *PIF1* and *SnRK3.9* were transcriptionally upregulated in response to REVd induction, supporting the idea that they are all bona fide target genes. We have previously shown that REV controls the expression of the class II HD-ZIP (HD-ZIPII) transcription factor genes *HAT2*, *HAT3*, *HAT4* and *ATHB4* (Brandt et al., 2012). Loss-of-function mutants in these *HD-ZIPII* genes result in strong leaf defects (Bou-Torrent et al., 2012) that are due to unregulated microRNA *MIR165/6* expression (Merelo et al., 2016). Here, we tested whether *HAT22*, which encodes another homeodomain leucine zipper protein, is also a direct REV target gene and we found that, as with the other candidates, *HAT22* expression was induced by REVd induction (Fig. S4). The latter finding is particularly important, as overexpression of *HAT22* has been shown to accelerate senescence (Kollmer et al., 2011). These results confirm previous findings and support a role for REV as a transcriptional activator that can induce expression of key regulators of diverse growth-regulating pathways.

### REV and MAX2 play partially antagonistic roles in vascular patterning

**Fig. 6.**
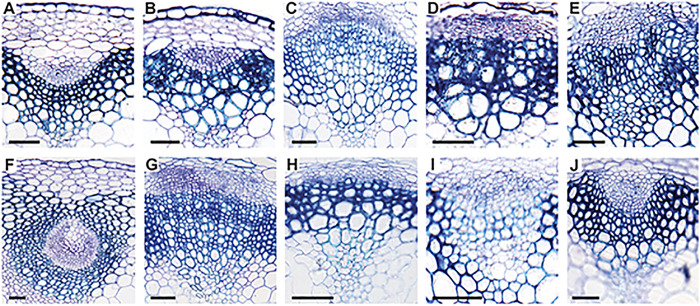
**Histological analysis of stem sections reveals partially antagonistic roles of REV and MAX2 in vascular patterning.** Stem cross-section of 6-week-old plants. Col-0 wild type (A), *max2-1* (B), *rev5* (C), *ZPR3-OX* (D), *MIR165a-OX* (E), *rev10D* (F), *max2-1 rev5* (G), *max2-1 ZPR3-OX* (H), *max2-1 MIR165a-OX* (I) and *max2-1 rev10D* (J). Scale bars: 50 µm.

Both REV and MAX2 control shoot patterning and loss-of-function mutations show the opposite phenotypes. REV is a known vascular regulator and it has been reported that *rev* gain-of-function mutants (*rev10D*) develop radial amphivasal vascular bundles (Emery et al., 2003). This prompted the analysis of whether *max2* mutant plants have altered vascular structures and whether there is a genetic relationship with regard to the vascular defects observed in *hd-zipIII* mutants. We sectioned the bases of the main stems of Col-0 wild type, and *max2* and *hd-zipIII* mutants, and respective *max2 hd-zipIII* double mutants, and examined morphologies of the vascular bundles. In comparison with Col-0 wild-type plants, *max2* mutant plants showed very similar vascular morphologies, except that the xylem parts contained an increased number of enlarged tracheary elements (Fig. 6A,B). Plants with loss-of-function mutations in *REV* (*rev5*) or transgenic plants that overexpress the *LITTLE ZIPPER3* gene or *MIR165a* all showed flattened vascular bundles (Fig. 6C-E), whereas bundles of *rev10D* plants were almost entirely radialized with xylem surrounding the phloem (Fig. 6F). The combinations of *max2* with *rev5* (*max2 rev5*), or transgenic plants that overexpress the *LITTLE ZIPPER3* (*max2 ZPR3-OX*) gene or *MIR165a* (*max2 MIR165a-OX*) resembled the respective *hd-zipIII* single mutants (Fig. 6G-I). The radial vascular bundles observed in *rev10D* mutant plants were, to a large degree, suppressed in *max2 rev10D* double mutant plants (Fig. 6J), indicating that either the REV10D-induced radialization requires a functional MAX2 protein or protein targets that are normally degraded by MAX2 accumulate and antagonize REV function. In agreement with this, we found that the known REV target genes *ZPR3*, *HOMEOBOX-LEUCINE ZIPPER PROTEIN 3* (*HAT3*) and *ARABIDOPSIS THALIANA HOMEOBOX-LEUCINE ZIPPER PROTEIN 4* (*ATHB4*) were slightly upregulated in the *max2* mutant background, indicative of MAX2 or MAX2 substrates influencing REV/HD-ZIPIII activity (Fig. S5A). We also assessed the expression of *REV* and several REV target genes in wild-type and transgenic *MAX2-OX* plants. While *REV* expression was slightly lower in plants overexpressing *MAX2*, we found increased levels of *ZPR3* and slightly lower levels of *HAT3* and *TAA1* (Fig. S5B). These findings indicate post-translational alterations in REV.

## DISCUSSION

To successfully grow and reproduce in a dynamic environment, plants evolved efficient signal perception and transduction systems. Downstream of the signal perception, transcriptional regulators dissipate the signal and convert it into transcriptional output. In that regard, several transcription factors can act as regulatory hubs and integrate signals from multiple pathways. Here, we report the identification of two signaling hubs regulating each other and thereby influencing diverse developmental processes. We found that the REV transcription factor physically interacted with the promoter of *MAX2*, and *rev10D* gain-of-function mutants showed elevated levels of *MAX2* expression. In agreement, the depletion of REV function or protein activity caused *MAX2* expression to decline in older leaves (Fig. 1C). This indicates that the regulation of *MAX2* is either stage specific or occurs in response to a specific signal. We have previously shown that the ability of REV to bind DNA *in vitro* is redox sensitive (Xie et al., 2014). It is therefore conceivable that the cellular redox-state of older leaves renders REV more active to regulate ‘late’ targets such as *MAX2*. Circumstantial support for this hypothesis is provided by a recent study on the role of the ZPR2 microProtein in regulating stem cell maintenance under hypoxia (Weits et al., 2019). The authors showed downregulation of many of the REV targets (including *MAX2*) upon *ZPR2* induction specifically under hypoxic conditions. Hypoxia and leaf senescence both affect the cellular redox state but likely through different pathways. In summary, our results support the notion that *MAX2* transcriptional regulation requires additional environmental inputs. Changes in redox state seem likely and it is imaginable that the PAS domain of REV acts as a redox sensor. Gain-of-function mutants in *rev* (here *rev10D*) had elevated levels of *MAX2* mRNA, which indicates that REV acts as an inducer of *MAX2* expression. A comparative study of the expression patterns of *REV*, *MAX2* and the known REV-target *ZPR3* revealed overlapping patterns of expression (Fig. 2), supporting the idea that *MAX2* is a direct target gene.

REV is a positive regulator of axillary meristem initiation. Loss-of-function *rev* mutants have a reduced number of axillary buds compared with *rev* gain-of-function mutants (*avb1*) that develop an increased number of axillary buds. On the contrary, MAX2 is a negative regulator of axillary bud outgrowth and loss-of-function mutants have a bushy appearance (Fig. 3). The finding that the combination of *rev* and *max2* mutants produced an intermediate phenotype implies that both factors partially operate in separate pathways. Side-shoot formation requires two steps: axillary bud formation and bud outgrowth. Here, REV promotes axillary bud formation (Otsuga et al., 2001) by promoting *STM* expression (Shi et al., 2016). In this study, we showed that REV also affected the subsequent bud outgrowth by regulating *MAX2*. Given that REV acts upstream of *MAX2* but MAX2 antagonizes REV function, there might be a negative-feedback mechanism involved in side shoot production: REV induces side shoots but the concomitant upregulation of a negative factor (MAX2) prevents excessive side shoot production. The fact that loss of REV function does not result in overall reduced expression levels of *MAX2* (Fig. 1) explains the barren appearance of *rev5* mutant plants. The finding that *hd-zipIII max2* double mutants (this includes *rev5 max2*, *ZPR3-OX max2* and *MIR165a-OX max2*) showed an intermediate phenotype suggests that *HD-ZIPIII* activity is required for the *max2* mutant phenotype and both factors control shoot branching in an interdependent manner.

*Arabidopsis* seedlings respond to shade by inducing elongation growth of the hypocotyl. Previous research has established a positive role for HD-ZIPIII transcription factors in promoting shade-induced growth (Baima et al., 2014; Brandt et al., 2012) and a negative role for MAX2 in repressing hypocotyl elongation (Shen et al., 2007). Similar to the control of shoot branching, we also found the combination of the *hd-zipIII* and *max2* mutants resulted in double-mutant plants that exhibited intermediate growth responses (Fig. 4). Some combinations, such as *max2 rev5*, showed elongated hypocotyls in white light and shade, and a partially restored shade-avoidance response, while *max2 ZPR3-OX* and *max2 MIR165a-OX* plants produced generally shorter hypocotyls; however, these plants were almost shade insensitive. In summary, these results show that the long hypocotyl phenotype of *max2* mutants is partially dependent on *HD-ZIPIII* activity, supporting a similar relationship to that previously observed in the control of shoot branching. Thus, HD-ZIPIIIs and MAX2 control shade-induced hypocotyl growth in an interdependent manner. MAX2 is a E3-ubiquitin ligase that potentially regulates many target substrates. It is possible that some of these target substrates directly or indirectly influence REV/HD-ZIPIII activity. The analysis of ChIP-seq and microarray data revealed that REV acts upstream of diverse growth-regulating pathways (Brandt et al., 2012; Merelo et al., 2013; Reinhart et al., 2013). Some of these regulations are positive and could promote context-dependent growth, whereas others would result in a context-dependent suppression of growth. A factor that, upon induction, would suppress growth is the brassinosteroid catabolism enzyme BAS1. The finding that REV acts as a direct and positive regulator of *BAS1* expression (Fig. S4) supports a rheostat-like function of REV: promoting growth but at the same time limiting excessive growth.

The onset of senescence initiates the last phase in the life of an annual plant. During this phase, carbon, mineral and nitrogen resources are remobilized from the senescing leaves to the reproductive organs and developing seeds; thus, senescence is important for the reproductive success, but can also be used as an exit strategy under harsh stress conditions. Senescence is mainly driven by transcriptional changes affecting more than 8000 genes during onset and progression (Breeze et al., 2011). Therefore, transcription factors play a pivotal role in this process. REV was already discovered as one of the factors regulating leaf senescence in a redox-dependent manner and thereby established a relationship between early and late leaf development (Xie et al., 2014). To date, the regulatory function of REV in senescence has been explained by its positive regulation of *WRKY53* expression, one of the central regulators of leaf senescence (Miao et al., 2004; Zentgraf et al., 2010). However, as the *rev5* senescence phenotype was more severe than the *wrky53* phenotype in this former study, it is now clear that REV targets additional SAGs (Xie et al., 2014). Here, we could show that one of these genes is *MAX2*. REV acts also upstream of *MAX2* and positively affects *MAX2* expression in some conditions. Accordingly, *max2* mutant plants show delayed senescence. The *rev5 max2* double mutant resembled *max2* mutants during early senescence and *rev5* mutants during late senescence, indicating that additive and independent effects exist and that they act in parallel. Surprisingly, *rev10D*, which contains a miRNA-insensitive version of REV and would therefore resemble a *REV*-overexpressing plant, shows only an accelerated senescence phenotype at early stages of senescence. This effect was negated in the absence of MAX2 (*max2 rev10D*), indicating that MAX2 is one of the most important downstream genes of REV in this phase. Overall, it was remarkable that the phenotype developed in two different phases: an early (5- and 6-week-old plants) and a late (8- and 9-week-old plants) phase. No significant difference was observed in 7-week-old plants in all lines compared with wild type, even though there is already a tendency towards a delay of senescence in almost all lines. Whereas in the late phase all lines showed significantly delayed senescence, in the early phase *max2 ZPR3-OX* and *rev10D*, in particular, showed significantly accelerated senescence, whereas *max2*, *max2 rev5* and *max2 rev10D* exhibit already delayed senescence, indicating an important but diverse function of MAX2 in early senescence. Taken together, our results reveal that there is no simple interplay between HD-ZIPIIIs, miRNA165a, LITTLE ZIPPER3 and MAX2, as a biphasic (early and late) development of senescence exists and different parts of the senescence program are touched in the different mutants. Most likely more players and more feedback loops exist in this complex regulatory expression network. Moreover, MAX2 might also regulate senescence through strigolactone signaling. *MAX2* encodes a F-Box protein, which can interact with the strigolactone receptor D14 and, upon hormone perception, leads to the degradation of a SMXL6/7/8-type repressor complex (Wang et al., 2015). Consistently, strigolactones can regulate leaf senescence in concert with ethylene in *Arabidopsis* (Ueda and Kusaba, 2015). Therefore, we can conclude that HD-ZIPIII proteins regulate senescence via WRKY53 and MAX2, and most likely other target genes, and that MAX2 is part of the complex multilevel network regulation of senescence influencing strigolacone signaling.

Growth responses, such as shade-induced elongation growth and shoot branching are controlled by REV and MAX2 in an interdependent fashion. In the senescence process, REV and MAX2 act synergistically, and with regard to vascular patterning it seems that MAX2 controls factors or processes that control REV activity (Fig. 6). This is supported by the finding that *rev10D* plants have strongly radialized vascular bundles compared with the bundles of *max2 rev10D* double mutant plants that resemble the wild type. To validate whether MAX2 controls unknown factors that influence REV protein activity, we tested the expression of three REV direct target genes in the *max2* mutant background. This analysis revealed that the expression levels of *ZPR3*, *HAT3* and *ATHB4*, three direct REV target genes (Brandt et al., 2012, 2013), were slightly upregulated in *max2* compared with wild-type plants (Fig. S5). This finding suggests feedback regulation of REV by MAX2, which could be direct or indirect. In the regulation of senescence, where REV and MAX2 have parallel functions, the loss of MAX2 might result in the upregulation of REV target genes and thereby enhance REV function. In summary, our findings show that different growth processes are controlled by REV and MAX2, but their regulatory hierarchy is dependent on the respective process that is likely a product of altered cellular environments (Table 1). Our results demonstrate that in order to achieve a holistic understanding of genetic regulation, a multilevel analysis is valuable, even though the results can turn out to be complex and difficult to understand.

## MATERIALS AND METHODS

### Plant material and growth conditions

All experiments were performed in the *Arabidopsis thaliana* Col-0 ecotype. The following mutant lines were used in this study: *max2-1* (Stirnberg et al., 2002), *rev5* (A260V), *rev10D* (gain-of-function allele), *ZPR3-OX* (Wenkel et al., 2007) and *MIR165a-OX* (Kim et al., 2010).

For all experiments, seeds were stratified for 3 days at 4°C in the dark before being plated on growth medium (GM) containing 4.3 g/l Murashige and Skoog medium (Duchefa), 10 g/l saccharose, 0.5 g/l MES and 5.5 g/l plant agar (Duchefa) (pH 5.8). The MS medium-grown and the soil-grown *Arabidopsis* plants were incubated under 100 µmol m^−2^ s^−1^ in a 16 h light/8 h dark cycle.

For senescence-phenotyping, *Arabidopsis thaliana* plant lines were grown in a climate chamber under long day conditions (16 h light, 8 h dark) with moderate light intensity (80-100 µE/m2/s) at an ambient temperature of 20°C. Individual leaves were labeled with threads of different color according to their age to identify the leaf age while harvesting within the first rosette even in late stages of senescence (Bresson et al., 2018). Leaves of the same position were used for the same specific analysis. Plants were harvested in a weekly rhythm and at the same time of the day to avoid circadian effects. Bolting and flowering occurred within 5 and 6 weeks, respectively.

For the GUS construct, a DNA fragment containing a 3001 bp upstream promoter region of MAX2 was PCR amplified using DNAs from Col-0 as templates. The resulting PCR product was cloned into pENTR/SD-TOPO vector and subsequently cloned into the pBGWFS7 vector to generate the proMAX2-GUS construct. The final constructs were introduced into Col-0 wild-type plants by an *Agrobacterium tumefaciens*-mediated floral dip. Transformed seeds were initially screened for resistance to the herbicide Basta, followed by confirmation by RT-PCR. Homozygous lines were selected and used for phenotypic analysis.

*In situ* hybridization was carried out with 12-day-old seedlings. *MAX2* probes were generated through amplifying of nucleotides 1 to 618 of the *MAX2*-coding sequence by PCR with the forward primer 5′-ATGGCTTCCACTACTCTCTC-3′ and reverse primer 5′-GCTTGATTTGTATCCCTCGG-3′.

### Chromatin-immunoprecipitation

For ChIP experiments and subsequent qPCR analysis, *35S::FLAG-GR-REVd* transgenic plants were grown in liquid MS medium supplemented with vitamins for 15 days in continuous light and induced with 25 μM dexamethasone (DEX) for 60 min. Samples were harvested and ChIP was carried out as described previously (Brandt et al., 2012).

### Hypocotyl measurements

For hypocotyl measurements, *Arabidopsis thaliana* plants were grown for 2 days in constant white light conditions to induce germination and then kept for another 4 to 5 days either in the same conditions or transferred to simulated canopy shade conditions.

### Senescence phenotyping

#### Senescence analysis

To investigate a difference in the progression of senescence between the plant lines, we performed a variety of methods that are described in detail by Bresson et al. (2018). Therefore, we harvested six plants (*n*=6) per plant line weekly, starting in the state before bolting at week 4 up to week 9, when leaves are heavily senescent. For the evaluation of leaf senescence phenotypes, rosette leaves were aligned according to their age with the help of color threads. Leaves of the first rosettes were aligned next to each other in order of their age. The automated colorimetric assay (ACA) defines leaf color pixel-wise and calculates the percentage of each category within the leaves, thereby providing information about chlorophyll degradation during senescence visible by a color change from green over yellow to brown. The activity of photosystem II (PSII) was assessed using a PAM chlorophyll fluorometer (Maxi version; version 2-46i, Heinz Walz). Therefore, Fv/Fm values were determined from leaf 5 and leaf 10. In addition, we measured the intracellular hydrogen peroxide level in leaf 8 using the fluorescent dye H_2_DCFDA. Electrolyte leakage was determined using a conductivity meter (CM100-2, Reid & Associates).

### qRT-PCR

QRT-PCR analysis was performed using senescence marker genes such as *SAG12* (AT5G45890), which encodes a cysteine protease, *SAG13* (*At2g29350*), which encodes a short-chain alcohol dehydrogenase, or *ORE1/ANAC092* (AT5G39610), a member of the NAC transcription factor family that plays a role in senescence. Expression was normalized to *ACTIN2*, relative quantification to either *GAPDH* (AT1G13440) or *ACTIN2* (At3g18780) was calculated using the ΔΔCT-method according to Pfaffl (Pfaffl, 2001). Total RNA was extracted using either the Spectrum Plant Total RNA Kit (Sigma) or the RNeasy Plant Mini Kit (Qiagen), followed by cDNA synthesis with RevertAid Reverse Transcriptase (Thermo Scientific). For the qPCR, KAPA SYBR Fast BioRad iCycler (KAPA Biosystems) master mix was used following the manufacturer's instruction.

### Axillary branching assay

The position of plants within flats was randomized to account for environmental variation. Total rosette branches at least 1 cm in length were counted for each plant at 6 weeks.

### Leaf morphology assay

The 7th leaf of each plant was marked with indelible marker at ∼3 weeks post-germination. The maximum length and width of the leaf blade were measured, as well as the length of the petiole.

### Histological analysis

Tissue of mutants and wild type were vacuumed for 15 min, fixed for 4 h in Karnovsky's Fixative, and embedded in resin, according to Spurr's procedure (Spurr, 1969). Sections (2 μm) were made on a SuperNova Reichert-Jung microtome, then stained with Toluidine Blue-O (0.05%, pH 4.4) and visualized in bright field using a Nikon Eclipse 80i Fluorescence microscope.

### GUS activity assay

The *REV::GUS* (*rev-9*), *MAX2::GUS* and *ZPR3::GUS* plants were incubated overnight in GUS staining solution [100 mM NaPO_4_ (pH 7.0), 10 mM EDTA, 0.5 mM K_4_Fe(CN)_6_, 0.5 mM K_3_Fe_6_, 0.01% Triton-X and 1 mM X-Gluc]. Samples were infiltrated under vacuum for 10 min and then incubated at 37°C overnight. The staining buffer was removed and the samples were cleared in 70% ethanol. All observations by light microscopy were made with the Nikon Eclipse 80i Fluorescence microscope.

### Accession numbers

Sequence data used in this article can be found in the Arabidopsis Genome Initiative or ENA databases under the following accession numbers: *MAX2* (AT2G42620), *REV* (AT5G60690), *ZPR3* (AT3G52770), *MIR165a* (AT1G01183), *SAG12* (AT5G45890), *SAG13* (AT2G29350), *ORE1/ANAC092* (AT5G39610), *BAS1* (AT2G26710), *GTL1* (AT1G33240), *HAT22* (AT4G37790), *HSL1* (AT1G28440), *PIF1* (AT2G20180), *SnRK3.9* (AT4G18700), *HAT3* (AT3G60390) and *ATHB4* (AT2G44910).

## Supplementary Material

Supplementary information

Reviewer comments
