# Supplementary material for: Multi-level analysis of the interactions between REVOLUTA and MORE AXILLARY BRANCHES 2 in controlling plant development reveals parallel, independent and antagonistic functions
Source: Development. 2020 May 21;147(10):dev183681. doi: 10.1242/dev.183681 (PMC7325436; doi:10.1242/dev.183681)
Supplement: Supplementary information [file develop-147-183681-s1.pdf]

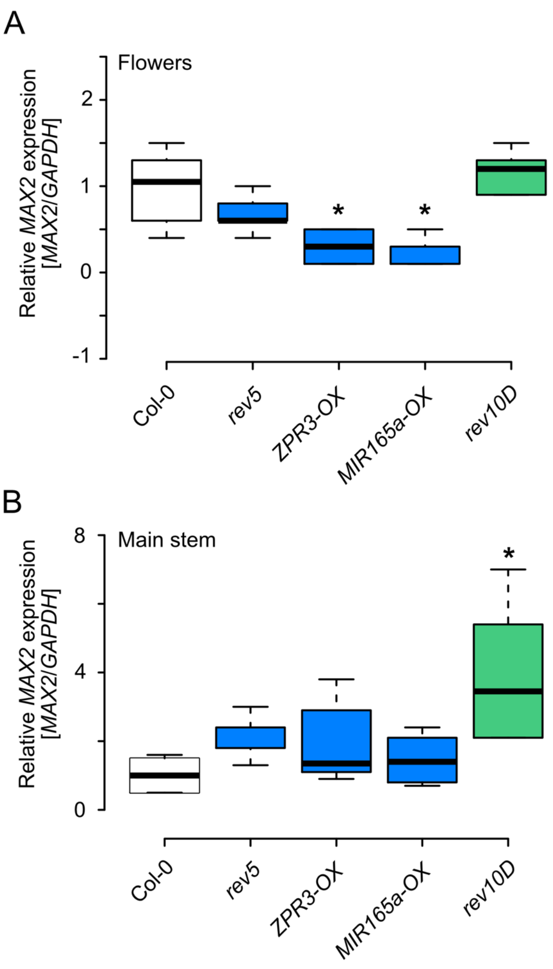

Figure S1 – Analysis of MAX2 expression in flowers and stems of wild type and mutant plants.

RT-qPCR analysis of *MAX2* expression in A, flowers and B, main stems of mature Arabidopsis plants. Expression of genes is relative to *GAPDH* which was used as internal reference gene. Plotted is the summary of two biological replicates. *P<0.05, **P<0,005***P<0.0005, Student's t-test.

Development • Supplementary information

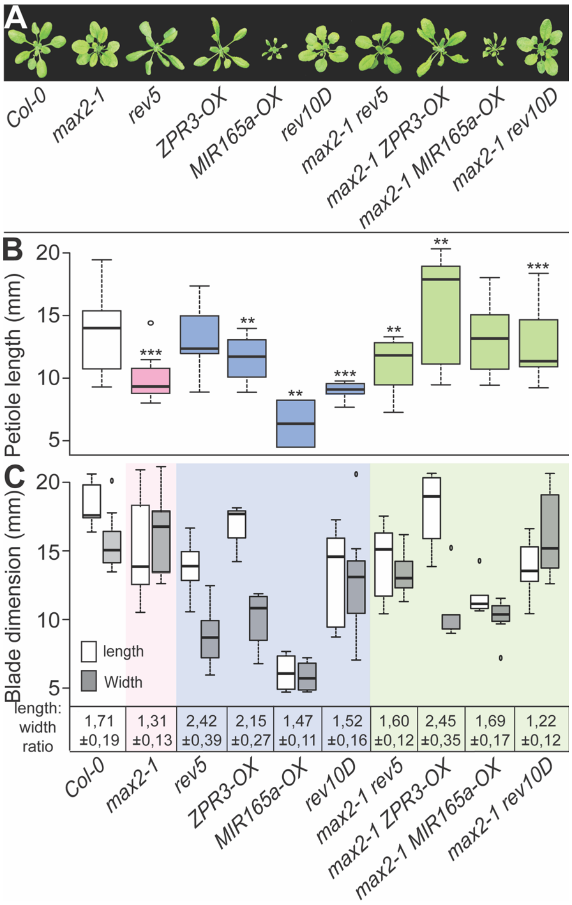

Figure S2 – MAX2 and HD-ZIPIIIs regulate different leaf traits.
(A) Phenotypes of rosettes of representative 3-week-old plants grown under LD photoperiod (16 h light/8 h dark). **(B)** Quantification of the length of petioles of the 7th leaf of 3-week-old of Col-0 (wildtype, white), single mutant plants (pink, blue), and double mutant plants (green) grown under LD photoperiod. **P<0.005, ***P<0.0005, Student's t-test. N=7-15. **(C)** Quantification of the length of the leaf blade (white) and width of the leaf blade (gray) of the 7th leaf of 3-week-old plants grown under LD photoperiod. Mean length:width ratio ±95% is shown below the x axis.

Development • Supplementary information

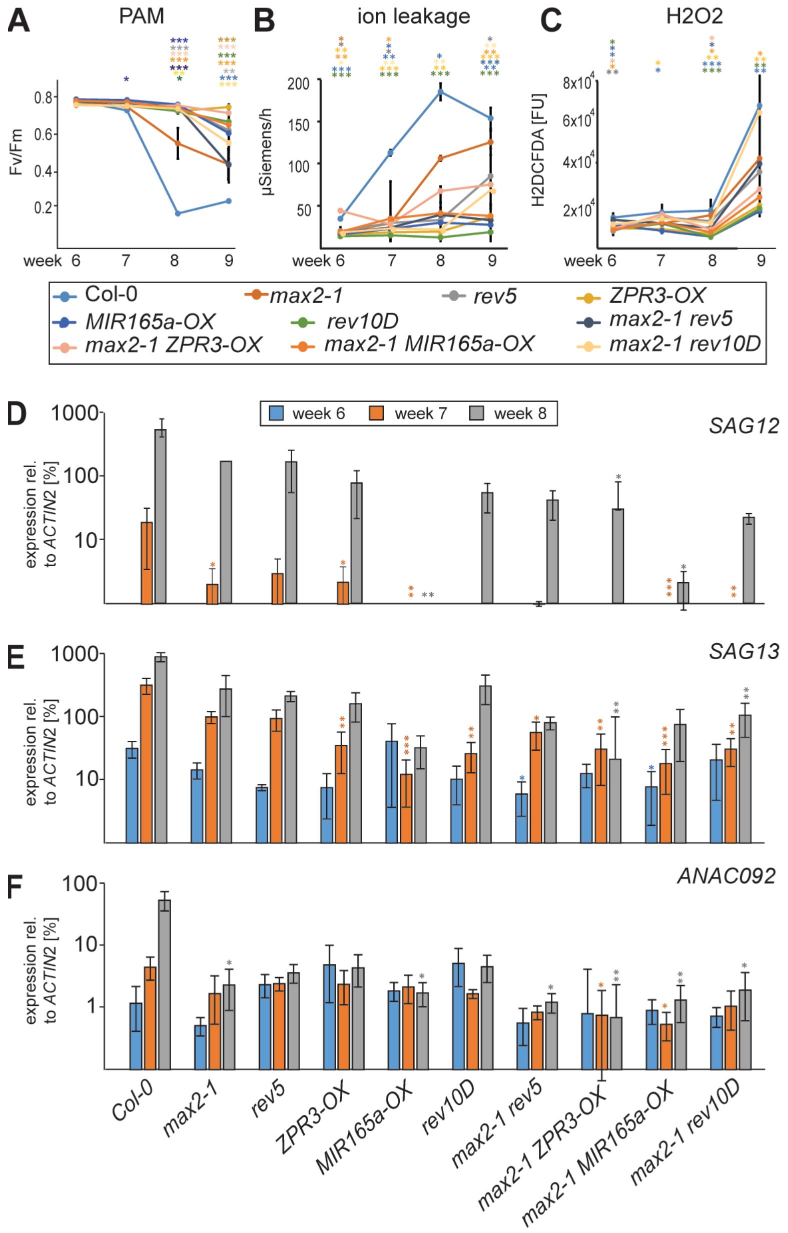

Figure S3 - Senescence phenotype parameters

Different key processes like photosynthetic capacities, membrane degradation, redox status and genetic regulation were analyzed as parameters involved in senescence. **(A)** The efficiency of photosystem II (Fv/Fm) was measured using the pulse amplitude modulation (PAM) chlorophyll fluorometer. Therefore, leaves at position 5 were analyzed. **(B)** Ion leakage over time was measured in single detached leaves at position 4 using a conductivity meter. **(C)** The H2O2 content was measured using carboxy-H2DCFDA fluorescence assay in independent leaves at position 9. Data are means (±SE) of 6 biological replicates of each line which were harvested weekly, shown here for week 6, 7, 8 and 9. Genetic regulation of senescence-related genes was analyzed by qRT-PCR using primers of different key markers like SAG12 **(D)**, SAG13 **(E)**, ANAC092 **(F)** qRT-PCR was done with a pool of 6 plants using either leaves at position 6 or 7. Expression levels were shown relative to *ACTIN2*. Data are means (±SE) n=3. Kruskal-Wallis-test was performed for statistically significant differences of all values at each timepoint compared to Col-0 (*$P \leq 0.05$, **$P \leq 0.01$, ***$P \leq 0.001$).

Development • Supplementary information

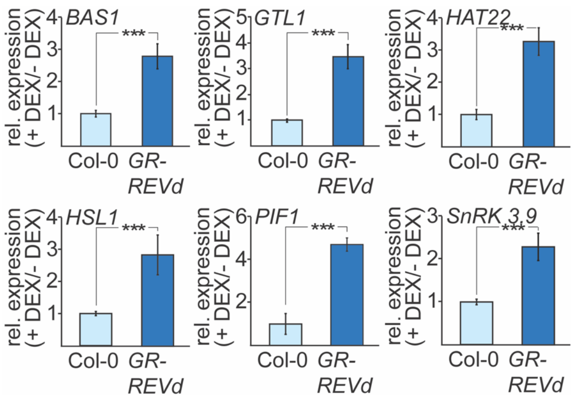

Figure S4 – REV regulates the expression of potential direct target genes involved in shoot branching/growth control.

Quantitative real-time PCR (RT-qPCR) experiments of putative REV target genes in 10-day-old Col-0 wild-type (light blue) and *35S::GR-REVd* (dark blue) seedlings in response to 60 min DEX (25µM) induction in the presence of the protein biosynthesis inhibitor cycloheximide (CHX). Plotted is the relative induction (+DEX +CHX/-DEX +CHX) for both genotypes as average values of three biological replicates ± SD. ***P<0.0005, Student's t-test.

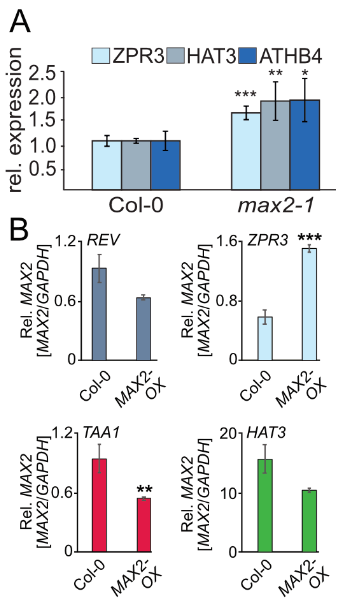

Figure S5 – Analysis of REV target genes in *max2* mutant plants suggests an antagonistic role of MAX2 to REV function.

(A) RT-qPCR analysis of REV direct downstream target genes (*ZPR3*, *HAT3*, and *ATHB4*) expression in 10-day-old seedlings of Col-0 and *max2-1*. Expression of genes is relative to *GAPDH* internal reference gene. Plotted is the summary of three biological replicates ± SD. *P<0.05, **P<0,005***P<0.0005, Student's t-test.

(B) RT-qPCR analysis of *REV*, *ZPR3*, *TAA1* and *HAT3* in wild type Col-0 and transgenic plants overexpressing *MAX2* (*MAX2-OX*, *35S::FLAG-MAX2*). **P<0,005***P<0.0005, Student's t-test.

Development • Supplementary information
