# Supplementary material for: Multi-level analysis of the interactions between REVOLUTA and MORE AXILLARY BRANCHES 2 in controlling plant development reveals parallel, independent and antagonistic functions
Source: Development. 2020 May 21;147(10):dev183681. doi: 10.1242/dev.183681 (PMC7325436; doi:10.1242/dev.183681)
Supplement: Reviewer comments [file develop-147-183681_review_history.pdf]

Multi-level analysis of the interactions between *REVOLUTA* and *MORE AXILLARY BRANCHES 2* in controlling plant development reveals parallel, independent and antagonistic functions

Shin-Young Hong, Esther Botterweg-Paredes, Jasmin Doll, Tenai Eguen, Anko Blaakmeer, Sanne Matton, Yakun Xie, Bjørg Skjøth Lunding, Ulrike Zentgraf, Chunmei Guan, Yuling Jiao and Stephan Wenkel
DOI: 10.1242/dev.183681

Editor: Yka Helariutta

Review timeline

Original submission:	9 August 2019
Editorial decision:	30 September 2019
First revision received:	11 December 2019
Editorial decision:	13 January 2020
Second revision received:	12 March 2020
Accepted:	19 April 2020

Original submission

First decision letter

MS ID#: DEVELOP/2019/183681

MS TITLE: Multi-level analysis of the interactions between REVOLUTA and MORE AXILLARY BRANCHES 2 in controlling plant development reveals parallel, independent and antagonistic functions

AUTHORS: Esther Botterweg, Shin-Young Hong, Jasmin Doll, Tenai Eguen, Anko Blaakmeer, Yakun Xie, Bjoerg Skjoeth Lunding, Ulrike Zentgraf, Yuling Jiao, and Stephan Wenkel

I have now received all the referees' reports on the above manuscript, and have reached a decision. The referees' comments are appended below, or you can access them online: please go to BenchPress and click on the 'Manuscripts with Decisions' queue in the Author Area.

As you will see, the referees express considerable interest in your work, but have some significant criticisms and recommend a substantial revision of your manuscript before we can consider publication. If you are able to revise the manuscript along the lines suggested, which may involve further experiments, I will be happy receive a revised version of the manuscript. Your revised paper will be re-reviewed by one or more of the original referees, and acceptance of your manuscript will depend on your addressing satisfactorily the reviewers' major concerns. Please also note that Development will normally permit only one round of major revision.

Please attend to all of the reviewers' comments and ensure that you clearly highlight all changes made in the revised manuscript. Please avoid using 'Tracked changes' in Word files as these are lost in PDF conversion. I should be grateful if you would also provide a point-by-point response detailing how you have dealt with the points raised by the reviewers in the 'Response to Reviewers' box. If you do not agree with any of their criticisms or suggestions please explain clearly why this is so.

© 2020. Published by The Company of Biologists under the terms of the Creative Commons Attribution License (https://creativecommons.org/licenses/by/4.0/).

Reviewer 1

Advance summary and potential significance to field

This paper investigates the roles of two important plant transcription factors REV and MAX2, in regulating a range of processes in Arabidopsis development including shoot branching, hypocotyl elongation and leaf senescence. The paper contains some meticulous and careful experiments involving quantification of mRNA levels, developmental growth assays of shoot branching and hypocotyl length, as well as senescence analysis admirably unpicking the progression of senescence through development in multiple ways. This would be of interest to researchers in multiple areas of plant development, such as those interested in shoot architecture, meristem regulation and hormone interactions.

Comments for the author

The majority of the experiments presented here have been carried out very well with a range of methods used to investigate multiple processes in plant development. The differences observed in different genotypes are quite clear and unambiguous. With some further consideration of the interpretation of the results, this work could be very good.

Although the paper makes an attempt to unpack the regulatory interactions between REV and MAX2, there are some issues with interpretation of these experiments, compromising the message of the manuscript.

Specifically, the authors carry out crosses and analyse a variety of Arabidopsis lines, largely those that reduce or increase the expression or protein stability of REV and MAX2. The rationale for this appears to be that REV is thought to directly regulate MAX2 expression, as shown by their ChIP-Seq analysis. Support for REV as a regulator of MAX2 expression is only actually confirmed in the gain-of-function rev10D mutant though. While this suggests that REV can regulate MAX2 in theory, it does not confirm that this interaction normally occurs. The authors attempt to address this limitation by demonstrating the overlapping expression patterns of REV and MAX2, but this is still quite circumstantial. It is possible that REV never, or only rarely actually regulates MAX2 expression in WT plants. As a result, many of the experiments that follow cannot be interpreted as relating to the direct interactions of MAX2 and REV, and so have limited relevance to the field in general.

A second issue is that the authors state that REV is an upstream regulator of MAX2 in the context of shoot branching. This is likely to be quite indirect however, as REV and MAX2 are involved in quite different aspects of shoot branching: REV regulates axillary meristem initiation, while MAX2 regulates axillary meristem elongation. The results presented here are consistent with completely independent roles for REV and MAX2. While this is addressed in the discussion, the description in the results (e.g. ‘REV and MAX2 have antagonistic roles in the regulation of shoot branching’) is misleading. REV and MAX2 are not necessarily acting antagonistically or necessarily interacting at all – they are likely to be acting on different processes.
Otherwise, the data here are well-presented and the analysis of senescence progression has been carried out particularly well. Multiple methods have been used to comprehensively assess different aspects of senescence.
If the authors can provide stronger evidence of the natural circumstances in which REV regulates MAX2 then this would be an excellent paper. As it is, it is difficult to place the experiments presented into a context relevant to other research.

Some minor points to additionally address:
- Statistical tests have been carried out for most data (except Fig S4) but not named or described for data in figure 1, 4, S1 and S3.
- For qPCR experiments, the tissue type/age is not mentioned in the manuscript.
- L82-87: confusing sentence, not grammatical
- L90: Missing ‘the’ in ‘In [the] case of’
- L97: The authors should cite Umehara et al 2008, Nature 455:195-200 as well as the Gomez-Roldan et al reference as the papers were published back to back.
- L98: The references are not correct here. The ‘second-messenger’ theory is not generally supported by Leyser lab work cited here. They argue that strigolactone acts by regulating auxin

© 2020. Published by The Company of Biologists under the terms of the Creative Commons Attribution License (https://creativecommons.org/licenses/by/4.0/).

transport. Additionally, the Waldie & Leyser paper is not about strigolactone action on auxin transport, it is about cytokinin.
- L154: ‘genetics’ should be ‘genetic’
- L175-179: some repetition in these sentences
- L255: confusing wording
- L292: strong statement – use of the word ‘pathways’ is a bit confusing as it implies that one is upstream of the other. The genes could be regulating senescence completely independently but share a common connection (directly or indirectly) with another regulator in the gene network.
- L451: this reference (Shi et al) is relevant but it is not the original paper showing a relationship between REV and STM. Otsuga et al 2001, Plant Journal 25: 223 – 226 is also relevant here.
- L472-3: confusing grammar/wording
- L507: ‘divers’ should be ‘diverse’ (though this is still a slightly odd word to use here)
- L572: senescence phenotyping description has ended up in the paragraph describing ChIP methods
- L607: statement about qPCR says expression is normalised to ACTIN2 but figure 1 and S4 show GAPDH normalisation.
- L614: For the axillary branching assay: given differences in senescence progression, is 6 weeks an equivalent developmental time point for different genotypes?

<u>Reviewer 2</u>

Advance summary and potential significance to field

The authors previously conducted a ChIPseq experiment to identify direct target genes of the transcription factor Revoluta (REV). Here, they analyzed one of these potential target genes, MAX2, an F-Box protein required for strigolactone signaling. They confirm by ChIPseq that REV binds the MAX2 promoter. MAX2 transcript levels were increased in a REV gain-of-function mutant expressing a miR-resistant REV suggesting that REV activates MAX2 expression. To address possible actions of REV and MAX2, the authors conducted a very elaborate double mutant analysis of max2 and rev genetic variants, analyzing a number of phenotypes: shoot branching, leaf shape and petiole length, shade avoidance response, leaf senescence and vascular patterning. Here, they find parallel, independent and antagonistic interactions of rev and max2.

The manuscript uncovers a possible regulation of MAX2 expression by REV. This is novel and of interest to many readers. The manuscript provides an interesting genetic analysis of double mutants. Also, a novel phenotype of the max2 mutant in the shade avoidance response to low R:FR was uncovered. The double mutant analysis remains rather descriptive, though, and it is not clear to what extent these genetic interactions relate to a regulation of MAX2 expression by REV. It does provide interesting information to scientists working in this specialty. The manuscript is well-written.

Comments for the author

Major comments:

1. The authors show that only gain-of-function but not loss-of-function mutations of REV affect MAX2 expression (Fig. 1C). They speculate that MAX2-regulation by REV is "either cell-type-specific or transient or occurs in response to a specific signal".
- This can in part be directly addressed, e.g. by RNA in situs in the meristem or by crossing of the available MAX2-promoter::GUS line (Fig. 2) into a rev mutant background.
- Also, other REV family members might be involved as well (PHV, PHB)? Is there redundancy and therefore no effect of rev loss-of-function? Has this been looked at?
- MAX2 and REV transcript levels co-express in a number of tissue types (flowers, meristems, vasculature root tips). Which tissue was used for the RT-qPCR analysis of MAX2 transcript levels in a rev mutant background (Fig. 1C)? Have other tissues been tested?
- does the gain-of-function allele of rev lead to ectopic expression of REV in other tissues or to overexpression in the normal expression domain?

© 2020. Published by The Company of Biologists under the terms of the Creative Commons Attribution License (https://creativecommons.org/licenses/by/4.0/).

2. The double mutant analysis remains rather descriptive and - in antagonistic interactions - does not agree with the observation that REVox increases MAX2 expression. This is also pointed out by the authors.
The authors suggest negative feedback loops, but this is rather speculative. It would be good to provide experimental evidence. To what extent do the results from the double mutant analysis indeed relate to a regulation of MAX2 expression by REV in the particular tissue?

Minor comments:

Fig. 4: The white and red boxes referring to WL and WL + FR appear to be missing in the figure.
Line 82 "In agreement with..." This sentence is unclear.

Reviewer 3

Advance summary and potential significance to field

The manuscript by Hong and Botterweg-Paredes et al. reports comprehensive investigations of the role of MORE AXILLARY BRANCHES 2 (MAX2) in the developmental regulation mediated by REVOLUTA. REVOLUTA (REV), a member of Class III homeodomain leucine zipper (HD-ZIPIII) transcription factors, plays important roles in various developmental processes. In prior studies, authors' lab reported genome-wide identification of REV binding sites and REV's involvement in the leaf senescence program via WRKY53. In this manuscript authors focused on MAX2 as direct target of REV based on ChIP data. Partly consistent with direct binding of REV on the MAX2 promoter, REV gain-of-function mutant, rev10D, showed upregulation of MAX2, while REV loss-of-function mutant, rev5 did not affect MAX2 expression. Authors analyzed shoot branching, leaf morphology, hypocotyl elongation in response to shade, leaf senescence, and vascular patterning in max2, rev5, rev10D, ZPR3-OX (condition suppressing HD-ZIP III activities), MIR165a-OX (condition reducing HD-ZIP III RNAs) lines, and combination of max2 and each of perturbations of REV. Through morphological analyses, authors conclude that REV and MAX2 in general antagonize each other, through complex genetic interactions.

Comments for the author

Overall, the manuscript provides comprehensive morphological analyses of Arabidopsis plants with various genetic combinations of MAX2 and REV mutants.
These will be informative to plant developmental biologists with a broad range of interest. At the same time, while covering many different aspects of development, authors could not clearly explain why they studied one developmental aspect and then the other.
The ChIP and quantitative RT-PCR data, which indicate MAX2 as direct downstream of REV, did not help understanding morphological data. Since the data indicate that rev10D increases MAX2 expression, one easily assumes that phenotype of rev10D would be masked by max2 mutant if MAX2 is a key downstream developmental regulator of REV. However, phenotypic analyses show that is not the case:
overall, the phenotype of rev10D was rather similar to max2 mutant's. Authors propose that is because MAX2 and REV play opposing roles in developmental processes being investigated. To me, the proposed model does not well fit with molecular data and needs further investigation, for example measuring REV in max2 mutant or ectopic MAX2 expressor to find whether there is an antagonizing role of MAX2 on REV.
It is clear that REV plays a role in multi-faceted developmental pathways. I hope authors could focus more on the pathways where molecular regulation between REV and MAX2, and morphological analyses support each other.

Specific comments:
Line 82:
In agreement with REV being as a positive regulator of shoot branching fail rev loss-of-function mutants to initiate axillary meristems and have a barren appearance
(revised to)
In agreement with REV being a positive regulator of shoot branching, rev loss-of-function mutants fail to initiate axillary meristems and have a barren appearance

© 2020. Published by The Company of Biologists under the terms of the Creative Commons Attribution License (https://creativecommons.org/licenses/by/4.0/).

Figure 1C.
What was the stage of plants and tissue/organs analyzed for quantitative RT-PCR? Are these data sufficient to show that rev5 does not affect basal MAX2 expression?

Line 179:
I think analyzing MAX2::GUS expression in rev loss- and/or gain-of-function mutant would be informative for explaining mutant phenotypes in the manuscript.

Line 226: delete ‘more’

Remarks on conclusion ending on line 270:
max2 hypocotyls elongate even under white light and this phenotype is slightly suppressed in max2 rev10D. I wonder MAX2 has a role in sensing FR while REV in responding to FR. In this case, they are not functioning in the same process.

Remark on section titled ‘REV regulates additional genes involved in shoot branching and growth control’:
The results in this section are no more than confirming the ChIP-seq results.
Considering there is no further genetic analyses, I do not think these additional data do not deepen the understanding of shoot branching process. I think this section can be combined with the first section and addressed in relation to MAX2.

Line 405: vessels -> bundles

Line 445: What is the difference between avb1 and rev10D? I think these alleles need to be explained to understand why their shoot branching patterns are different.

Line 455: The fact that loss of REV function does not resulted in reduced expression of MAX2 expression (revise to)
The fact that loss of REV function does not result in reduced expression of MAX2

Line 549: FLAG-MAX -> do you mean FLAG-REV? and check the rest of the section

---

First revision

Author response to reviewers' comments

Reviewer 1 Advance Summary and Potential Significance to Field:
This paper investigates the roles of two important plant transcription factors, REV and MAX2, in regulating a range of processes in Arabidopsis development, including shoot branching, hypocotyl elongation and leaf senescence. The paper contains some meticulous and careful experiments involving quantification of mRNA levels, developmental growth assays of shoot branching and hypocotyl length, as well as senescence analysis admirably unpicking the progression of senescence through development in multiple ways. This would be of interest to researchers in multiple areas of plant development, such as those interested in shoot architecture, meristem regulation and hormone interactions.

Response: We appreciate the reviewer’s interest in our manuscript especially embracing its comprehensiveness by addressing multiple research areas.

Reviewer 1 Comments for the Author:
The majority of the experiments presented here have been carried out very well with a range of methods used to investigate multiple processes in plant development. The differences observed in different genotypes are quite clear and unambiguous. With some further consideration of the interpretation of the results, this work could be very good.

© 2020. Published by The Company of Biologists under the terms of the Creative Commons Attribution License (https://creativecommons.org/licenses/by/4.0/).

Although the paper makes an attempt to unpack the regulatory interactions between REV and MAX2, there are some issues with interpretation of these experiments, compromising the message of the manuscript.

Specifically, the authors carry out crosses and analyse a variety of Arabidopsis lines, largely those that reduce or increase the expression or protein stability of REV and MAX2. The rationale for this appears to be that REV is thought to directly regulate MAX2 expression, as shown by their ChIP-Seq analysis. Support for REV as a regulator of MAX2 expression is only actually confirmed in the gain-of-function rev10D mutant though. While this suggests that REV can regulate MAX2 in theory, it does not confirm that this interaction normally occurs. The authors attempt to address this limitation by demonstrating the overlapping expression patterns of REV and MAX2, but this is still quite circumstantial. It is possible that REV never, or only rarely, actually regulates MAX2 expression in WT plants. As a result, many of the experiments that follow cannot be interpreted as relating to the direct interactions of MAX2 and REV, and so have limited relevance to the field in general.

Response: The reviewer is correct in pointing out that the correlations between gene expression and the observed phenotypic changes are weak and appear circumstantial. As described above, we analyzed respective genetic interactions in various physiological pathways. However, one limitation in linking phenotypes to gene expression changes could be that the major regulation of *MAX2* might not occur at the transcriptional level hence it may be difficult to prove. We have addressed this shortcoming by analyzing *MAX2* expression 1) in leaves of young and old plants
2) in other tissues (here flowers and stems of mature plants).
We found that in young leaves expression of *MAX2* is only induced in the *rev10D* background (previous data) but in 4-week-old leaves we found a significant reduction of *MAX2* in *rev5*, *35S::ZPR3* and *35S::miR165a* compared to wild type plants of the same age. This indicates that the regulation of *MAX2* by REV is especially relevant at later stages of development. Furthermore, this could also indicate that REV might be regulated by the cellular redox state. This would agree with our previous findings that REV binds DNA in a redox-sensitive manner (Xie et al., Development 2014). Additional support comes from a recent study on the role of the ZPR2 protein in stem cell maintenance under hypoxia (Weits et al., Nature Vol. 569, 2019). In this study the authors showed downregulation of many of the REV targets (including MAX2) upon ZPR2 induction specifically under hypoxic conditions further supporting the notion that *MAX2* transcriptional regulation requires additional environmental inputs. We have updated both the results and discussion.
The analysis of *MAX2* expression levels in different tissues revealed no change of *MAX2* in stems of plants with reduced REV/HD-ZIPIII levels but an increase of *MAX2* expression in *rev10D*. The analysis of MAX2 levels in flowers revealed significantly lower levels of MAX2 in flowers of plants overexpressing either ZPR3 or MIR165A. Taken together, these data indicate that the regulation of *MAX2* by REV is highly context-dependent.

A second issue is that the authors state that REV is an upstream regulator of MAX2 in the context of shoot branching. This is likely to be quite indirect, however, as REV and MAX2 are involved in quite different aspects of shoot branching: REV regulates axillary meristem initiation, while MAX2 regulates axillary meristem elongation. The results presented here are consistent with completely independent roles for REV and MAX2. While this is addressed in the discussion, the description in the results (e.g. 'REV and MAX2 have antagonistic roles in the regulation of shoot branching') is misleading. REV and MAX2 are not necessarily acting antagonistically or necessarily interacting at all – they are likely to be acting on different processes. Otherwise, the data here are well-presented and the analysis of senescence progression has been carried out particularly well. Multiple methods have been used to comprehensively assess different aspects of senescence. If the authors can provide stronger evidence of the natural circumstances in which REV regulates MAX2 then this would be an excellent paper. As it is, it is difficult to place the experiments presented into a context relevant to other research.

Response: We wish to thank this reviewer for her/his encouraging comments. We agree that REV and MAX2 might have independent roles in the control of shoot branching. We have edited the manuscript accordingly making this point clearer.

Some minor points to additionally address:

© 2020. Published by The Company of Biologists under the terms of the Creative Commons Attribution License (https://creativecommons.org/licenses/by/4.0/).

- Statistical tests have been carried out for most data (except Fig S4) but not named or described for data in figure 1, 4, S1 and S3.

Response: The reviewer is correct and we have edited respective figure legends.

- For qPCR experiments, the tissue type/age is not mentioned in the manuscript.

Response: We have added this information to the respective figure legend.

- L82-87: confusing sentence, not grammatical

Response: We have amended respective sentence.

- L90: Missing 'the' in 'In [the] case of'

Response: We have inserted the missing word.

- L97: The authors should cite Umehara et al 2008, Nature 455:195-200 as well as the Gomez Roldan et al reference as the papers were published back to back.

Response: We apologize for the carelessness and have included the respective citation.

- L98: The references are not correct here. The 'second-messenger' theory is not generally supported by Leyser lab work cited here. They argue that strigolactone acts by regulating auxin transport. Additionally, the Waldie & Leyser paper is not about strigolactone action on auxin transport, it is about cytokinin.

Response: We apologize for the inadvertence and have revised respective section.

- L154: 'genetics' should be 'genetic'

Response: We corrected this typo.

- L175-179: some repetition in these sentences

Response: We have edited respective sentences.

- L255: confusing wording

Response: We have amended respective sentence.

- L292: strong statement – use of the word 'pathways' is a bit confusing as it implies that one is upstream of the other. The genes could be regulating senescence completely independently but share a common connection (directly or indirectly) with another regulator in the gene network.

Response: We have amended respective sentence and instead of simply referring to "pathways" we refer to "physiological pathways".

- L451: this reference (Shi et al) is relevant but it is not the original paper showing a relationship between REV and STM. Otsuga et al 2001, Plant Journal 25: 223 – 226 is also relevant here.

Response: We have included respective reference.

- L472-3: confusing grammar/wording

Response: We have edited the sentence.

- L507: 'divers' should be 'diverse' (though this is still a slightly odd word to use here)

Response: We have corrected the typo.

- L572: senescence phenotyping description has ended up in the paragraph describing ChIP methods

Response: We have moved respective paragraph to the 'Plant Material and Growth Conditions' section.

- L607: statement about qPCR says expression is normalised to ACTIN2 but figure 1 and S4 show GAPDH normalisation.

Response: Some qPCR experiments were carried out in Tübingen where ACTIN2 is routinely used as reference gene while the Copenhagen lab routinely uses GAPDH. We have edited the material and

© 2020. Published by The Company of Biologists under the terms of the Creative Commons Attribution License (https://creativecommons.org/licenses/by/4.0/).

methods part accordingly.

- L614: For the axillary branching assay: given differences in senescence progression, is 6 weeks an equivalent developmental time point for different genotypes?

Response: That is a valid point and we have analyzed later timepoints. However, the results obtained after six weeks are not markedly different from the later timepoints. For this reason, we only show the six week results.

Reviewer 2 Advance Summary and Potential Significance to Field:
The authors previously conducted a ChIPseq experiment to identify direct target genes of the transcription factor Revoluta (REV). Here, they analyzed one of these potential target genes, MAX2, an F-Box protein required for strigolactone signaling. They confirm by ChIPseq that REV binds the MAX2 promoter. MAX2 transcript levels were increased in a REV gain-of- function mutant expressing a miR-resistant REV, suggesting that REV activates MAX2 expression. To address possible actions of REV and MAX2, the authors conducted a very elaborate double mutant analysis of max2 and rev genetic variants, analyzing a number of phenotypes: shoot branching, leaf shape and petiole length, shade avoidance response, leaf senescence and vascular patterning. Here, they find parallel, independent and antagonistic interactions of rev and max2.

The manuscript uncovers a possible regulation of MAX2 expression by REV. This is novel and of interest to many readers. The manuscript provides an interesting genetic analysis of double mutants. Also, a novel phenotype of the max2 mutant in the shade avoidance response to low R:FR was uncovered. The double mutant analysis remains rather descriptive, though, and it is not clear to what extent these genetic interactions relate to a regulation of MAX2 expression by REV. It does provide interesting information to scientists working in this specialty. The manuscript is well-written.

Response: We thanks this reviewer for her/his encouraging comments.

Reviewer 2 Comments for the Author:
Major comments:

1. The authors show that only gain-of-function but not loss-of-function mutations of REV affect MAX2 expression (Fig. 1C). They speculate that MAX2-regulation by REV is "either cell-type- specific or transient or occurs in response to a specific signal".

- This can in part be directly addressed, e.g. by RNA in situs in the meristem or by crossing of the available MAX2-promoter::GUS line (Fig. 2) into a rev mutant background.

Response: We have addressed this issue. Please read our response to the first major comment of reviewer #1. Crossing the MAX2::GUS lines with respective mutants would take considerable time. For this reason, we now assessed *MAX2* expression in wild type, *rev5* and *rev10D* by *in situ* hybridization. Although signals are quite weak, we do see *MAX2* expression more towards the adaxial domain (in which *REV* is expressed). Furthermore, in *rev10D*, *MAX2* expression is patchier and extends towards the abaxial domain. These data support a role of REV as an upstream regulator of *MAX2*.

- Also, other REV family members might be involved as well (PHV, PHB)? Is there redundancy and therefore no effect of rev loss-of-function? Has this been looked at?

Response: This is a very valid point. The *phb phv rev* triple mutant (published by Mike Prigge and Steven Clark see Plant Cell 17(1):61-76, 2005) arrests very early in development and for these reasons we analyzed transgenic plants overexpressing the *LITTLE ZIPPR3* gene or *MIR165a*, that affect HD-ZIPIII activity more globally. However, *MAX2* expression is only marginally affected in two-week old plants. As outlined in our response to reviewer #1, we now analyzed *MAX2* levels in older (4-week old) plants and observed significantly lower levels of *MAX2* in *rev5*, *ZPR3-OX* and *MIR165A-OX*, supporting the role of REV as a direct regulator. In addition, we also detected lower level of MAX2 in flowers of *ZPR3-OX* and *MIR165A-OX* plants (new Figure S1). In summary, we conclude that the regulation of *MAX2* by REV is highly context-dependent.

- MAX2 and REV transcript levels co-express in a number of tissue types (flowers, meristems, vasculature, root tips). Which tissue was used for the RT-qPCR analysis of MAX2 transcript levels in

© 2020. Published by The Company of Biologists under the terms of the Creative Commons Attribution License (https://creativecommons.org/licenses/by/4.0/).

a rev mutant background (Fig. 1C)? Have other tissues been tested?
Response: This experiment was done with leaves of two-week old plants. We have edited the figure legend accordingly and now also include 4-week old plants and as outlined above analyzed flowers and stems (Figure S1).

- does the gain-of-function allele of rev lead to ectopic expression of REV in other tissues or to overexpression in the normal expression domain?
Response: This is an interesting question worth examining but in this study, we have not checked where and when REV is expressed in this mutant background. We speculate that its expression domain is expanded due to the transformative nature of the HD-ZIPIII proteins. This has been observed for other HD-ZIPIIIs (see McConnell et al., Nature 2001). Given the size of the REV protein it appears unlikely that it can move out of its mRNA expression domain.

2. The double mutant analysis remains rather descriptive and - in antagonistic interactions - does not agree with the observation that REVox increases MAX2 expression. This is also pointed out by the authors. The authors suggest negative feedback loops, but this is rather speculative. It would be good to provide experimental evidence. To what extent do the results from the double mutant analysis indeed relate to a regulation of MAX2 expression by REV in the particular tissue?
Response: We tested the expression of *REV* and three REV-targets (*TAA1*, *ZPR3* and *HAT3*) in transgenic plants overexpressing *MAX2* (Fig. S5). In these experiments we found that *REV* expression is slightly lower in *MAX2-OX* plants but several of REV targets have strongly altered levels of expression. Suggesting post-translational changes downstream of REV.

Minor comments:

Fig. 4: The white and red boxes referring to WL and WL + FR appear to be missing in the figure.
Response: The red and white boxes refer to the box plots. We have amended the legend making this clearer.

Line 82 "In agreement with..." This sentence is unclear.
Response: We have amended respective sentence.

Reviewer 3 Advance Summary and Potential Significance to Field:
The manuscript by Hong and Botterweg-Paredes et al. reports comprehensive investigations of the role of MORE AXILLARY BRANCHES 2 (MAX2) in the developmental regulation mediated by REVOLUTA. REVOLUTA (REV), a member of Class III homeodomain leucine zipper (HD-ZIPIII) transcription factors, plays important roles in various developmental processes. In prior studies, authors' lab reported genome-wide identification of REV binding sites and REV's involvement in the leaf senescence program via WRKY53. In this manuscript, authors focused on MAX2 as direct target of REV based on ChIP data. Partly consistent with direct binding of REV on the MAX2 promoter, REV gain-of- function mutant, rev10D, showed upregulation of MAX2, while REV loss-of- function mutant, rev5 did not affect MAX2 expression. Authors analyzed shoot branching, leaf morphology, hypocotyl elongation in response to shade, leaf senescence, and vascular patterning in max2, rev5, rev10D, ZPR3-OX (condition suppressing HD-ZIP III activities), MIR165a-OX (condition reducing HD-ZIP III RNAs) lines, and combination of max2 and each of perturbations of REV. Through morphological analyses, authors conclude that REV and MAX2 in general antagonize each other, through complex genetic interactions.

Reviewer 3 Comments for the Author:
Overall, the manuscript provides comprehensive morphological analyses of Arabidopsis plants with various genetic combinations of MAX2 and REV mutants. These will be informative to plant developmental biologists with a broad range of interest. At the same time, while covering many different aspects of development, authors could not clearly explain why they studied one developmental aspect and then the other. The ChIP and quantitative RT-PCR data, which indicate MAX2 as direct downstream of REV, did not help understanding morphological data. Since the data indicate that rev10D increases MAX2 expression, one easily assumes that phenotype of rev10D would be masked by max2 mutant if MAX2 is a key downstream developmental regulator of REV. However, phenotypic analyses show that is not the case: overall, the phenotype of rev10D was rather similar to max2 mutant's. Authors propose that is because MAX2 and REV play opposing roles in

© 2020. Published by The Company of Biologists under the terms of the Creative Commons Attribution License (https://creativecommons.org/licenses/by/4.0/).

developmental processes being investigated. To me, the proposed model does not well fit with molecular data and needs further investigation, for example measuring REV in max2 mutant or ectopic MAX2 expressor to find whether there is an antagonizing role of MAX2 on REV. It is clear that REV plays a role in multi-faceted developmental pathways. I hope authors could focus more on the pathways where molecular regulation between REV and MAX2, and morphological analyses support each other.
Response: We appreciate the reviewer's interest in our work and her/his valuable suggestions to improve our manuscript. We agree that the data does not point in one particular direction, hence the title. To obtain a more holistic picture on the interaction between REV and MAX2, we investigated all physiological pathways they are involved in.

Specific comments:
Line 82:
In agreement with REV being as a positive regulator of shoot branching fail rev loss-of-function mutants to initiate axillary meristems and have a barren appearance (revised to)
In agreement with REV being a positive regulator of shoot branching, rev loss- of-function mutants fail to initiate axillary meristems and have a barren appearance
Response: We have revised respective sentence.

Figure 1C.
What was the stage of plants and tissue/organs analyzed for quantitative RT- PCR? Are these data sufficient to show that rev5 does not affect basal MAX2 expression?
Response: The experiment was carried out with leaves of two-week old plants. We have amended the legend accordingly and added additional data from 4-week-old plants.

Line 179:
I think analyzing MAX2::GUS expression in rev loss- and/or gain-of-function mutant would be informative for explaining mutant phenotypes in the manuscript.
Response: We agree with reviewer that this would be informative but unfortunately this material is currently not available and producing this material would take at least six months.

Line 226: delete 'more'
Response: We have deleted the respective word.

Remarks on conclusion ending on line 270:
max2 hypocotyls elongate even under white light and this phenotype is slightly suppressed in max2 rev10D. I wonder MAX2 has a role in sensing FR while REV in responding to FR. In this case, they are not functioning in the same process.
Response: We know that REV acts as a positive regulator of transcription factors involved in the shade avoidance response and of genes encoding auxin biosynthesis enzymes. Thus, the *rev*- phenotype is consistent with disturbed shade signaling. The phenotype of *max2-1* certainly resembles a constitutively active mutant (such as *phyB*). The substrates of MAX2 are on the other hand not known, which makes it difficult to speculate which step of the process is affected (perception vs signaling).

Remark on section titled 'REV regulates additional genes involved in shoot branching and growth control':
The results in this section are no more than confirming the ChIP-seq results. Considering there is no further genetic analyses, I do not think these additional data do not deepen the understanding of shoot branching process. I think this section can be combined with the first section and addressed in relation to MAX2.
Response: The reviewer is right in pointing out that the respective section does not provide further genetic analysis. However, we feel that merging this section with the first section would disturb the flow because we only establish in the later sections the effects on shoot branching and growth. For these reasons we would prefer not to merge the sections.

Line 405: vessels -> bundles
Response: We change the word.

© 2020. Published by The Company of Biologists under the terms of the Creative Commons Attribution License (https://creativecommons.org/licenses/by/4.0/).

Line 445: What is the difference between avb1 and rev10D? I think these alleles need to be explained to understand why their shoot branching patterns are different.
Response: Thanks for pointing this out and I looked the information up. Interestingly, *avb1* and *rev10D* are identical. They both change the proline at position 190 to leucine (P190L) but were isolated in independent screens (Zhong and Ye, Plant Cell Physiol. 45(4): 369–385, 2004 and Emery and Bowman, Current Biology, Vol. 13, 1768–1774, 2003). We worked with *rev10D* from John Bowman's group and introgressed it into the Col-0 background. We have not studied the *avb1* allele and it could well be that the difference in phenotype is a result of additional modifiers in the *avb1* background.

Line 455: The fact that loss of REV function does not resulted in reduced expression of MAX2 expression 1(revise to)
The fact that loss of REV function does not result in reduced expression of MAX2
Response: We have changed the wording accordingly.

Line 549: FLAG-MAX -> do you mean FLAG-REV? and check the rest of the section
Response: Thanks for pointing this out. We removed the erroneous text passage.

Second decision letter

MS ID#: DEVELOP/2019/183681

MS TITLE: Multi-level analysis of the interactions between REVOLUTA and MORE AXILLARY BRANCHES 2 in controlling plant development reveals parallel, independent and antagonistic functions

AUTHORS: Esther Botterweg, Shin-Young Hong, Jasmin Doll, Tenai Eguen, Anko Blaakmeer, Sanne Matton, Yakun Xie, Bjoerg Skjoeth Lunding, Ulrike Zentgraf, Chunmei Guan, Yuling Jiao, and Stephan Wenkel

I have now received all the referees' reports on the above manuscript, and have reached a decision. The referees' comments are appended below, or you can access them online: please go to BenchPress and click on the 'Manuscripts with Decisions' queue in the Author Area.

As you will see, the referees continue to express considerable interest in your work, but they still have some significant criticisms and recommend a substantial revision of your manuscript before we can consider publication. If you are able to revise the manuscript along the lines suggested, which may involve further experiments, I will be happy receive a revised version of the manuscript. Your revised paper will be re-reviewed by one or more of the original referees, and acceptance of your manuscript will depend on your addressing satisfactorily the reviewers' major concerns. Please also note that Development will normally permit only one round of major revision.

Please attend to all of the reviewers' comments and ensure that you clearly highlight all changes made in the revised manuscript. Please avoid using 'Tracked changes' in Word files as these are lost in PDF conversion. I should be grateful if you would also provide a point-by-point response detailing how you have dealt with the points raised by the reviewers in the 'Response to Reviewers' box. If you do not agree with any of their criticisms or suggestions please explain clearly why this is so.

Reviewer 1

Advance summary and potential significance to field

As before, this paper addresses potential interactions between two important plant developmental regulators, REV/HD-ZIPIIIs and MAX2. A range of experiments have been carried out to look at the

© 2020. Published by The Company of Biologists under the terms of the Creative Commons Attribution License (https://creativecommons.org/licenses/by/4.0/).

extent to which REV and MAX2 regulated similar aspects of plant development, including gene expression analysis, shoot branching, hypocotyl elongation and senescence progression.

Comments for the author

The revised paper by Hong, Botterweg-Paredes et al has a number of changes to the text and figures, some of which have been highlighted by the authors. Most of the minor points originally raised have been adequately addressed and an attempt has been made to address the major points. The additional experiments and changes to the text have been partially successful in correcting the previous issues, but there are still some concerns.

1) In their response to the reviewers, the authors state that an interaction between REV and MAX2 may not occur via transcriptional regulation.
It is, of course, true that regulatory interactions may not occur at the transcriptional level, however transcriptional level interactions are the evidence provided in this paper and used as a premise for experiments that follow in the manuscript. It is certainly possible that protein-level interactions occur, but there is no data provided to support that here. If transcriptional regulations cannot be fully relied upon and there is no protein level evidence, then it is hard to understand the rationale for the remainder of the paper. Despite this statement, however, the authors have now provided some additional evidence for the transcriptional level regulations by examining MAX2 transcripts in REV loss of function backgrounds. These provide some evidence for a fairly modest change in gene expression in certain tissues.

The authors also provide in situ data, though I do not find the images very convincing. All images provided appear pretty similar in expression and there does not seem to be any decrease in rev5 compared to wild type. The MAX2 RNA levels in rev10d seem very similar in the leaf primordia to WT and rev5 (comparing p3 with p3, p4 with p4 etc). The only place where there is an apparent increase is in the central structure between the primordia (the meristem perhaps?) This structure is not present in the other images so it is hard to make a comparison. Is this ectopic expression of MAX2 in rev10d? With more appropriate comparative images it would be easier to evaluate this.

Overall, the new data presented partially address the original issue (that rev10d plants show more dramatic MAX2 expression changes than loss of function mutants) by identifying some circumstances in which rev/HDZIP loss of function plants sometimes exhibit reduced MAX2 expression. However, the data presented do not fully rule out the idea that many of the rev10d phenotypes may be due to ectopic expression (such as in young leaves) and may not play very significant roles in normal development.

2) In conjunction with the previous point, there is some confusion in the manuscript over the nature of the proposed regulations between MAX2 and REV.
Specifically, the authors state in some cases that developmental processes are independent, but also state that their data support REV regulation of MAX2 in these aspects of development. For example, lines 218-221 and lines 487-496.

Some of the lack of clarity in this paper, appears to arise from conflict over whether the proposed interactions are direct or indirect. Both possibilities are potentially interesting but in different ways. Direct interactions imply a key regulatory relationship between important genes. Indirect interactions indicate that complex signaling networks in developmental processes are interconnected as parts of entire networks may overlap. With this in mind complex interconnected networks with multiple feedback loops may explain some of the unexpected apparently antagonistic interactions.
It would be helpful to establish clearly somewhere in the manuscript which developmental phenotypes likely occur as a result of direct interactions between REV and MAX2, indirect interactions, or no interaction.

There is a lot of good data in this paper, but it lacks a coherent interpretation. Some additional work on these aspects could be very helpful.

Some minor typos:
Line 83: ‘abv1’ typo Line 89: extra ‘as’

© 2020. Published by The Company of Biologists under the terms of the Creative Commons Attribution License (https://creativecommons.org/licenses/by/4.0/).

Line 91: extra ‘is’
Line 100: ‘which’ should be replaced with ‘that’
Line 101: missing comma after pathway Line 103: ‘cytokinine’ typo Line 204-205: ‘This contrasts the role…’ – unclear what this sentence means Line 237: extra ‘more’
Line 352: ‘In constistence with’ should be ‘Consistent with’
Line 437: ‘flatted’ should be ‘flattened’
Line 568: This doesn’t ‘contradict’ the REV regulation by MAX2 – it just implies that there may be feedback Line 692: The contributions of one of the new authors has not been described

Reviewer 2

Advance summary and potential significance to field

Please see my comment in 1st submission.

Comments for the author

In the revised version, the authors addressed some of the issues but did not provide sufficient evidence that REV indeed regulates MAX2 expression. Moreover, this shortcoming is not sufficiently presented in the manuscript: e.g. it is not mentioned in the abstract.

The results of the extensive double mutant analyses are still very interesting to the scientific community. However, either additional experiments showing reduced MAX2 levels in rev loss-of-function mutants are needed (e.g. by further addressing the REV family redundancy issue or using the MAX2-promoter::GUS lines) or a more balanced presentation and interpretation of the data is recommended, thereby clearly outlining the limitations of the analysis.

Reviewer 3

Advance summary and potential significance to field

The revised manuscript by Hong and Botterweg-Paredes et al. reports comprehensive investigations of the genetic interaction between MORE AXILLARY BRANCHES 2 (MAX2) and REVOLUTA (REV), a member of Class III homeodomain leucine zipper (HD-ZIPIII) transcription factors, in various developmental processes. Though REV protein can directly bind to the promoter of MAX2, their interaction in diverse developmental processes seems to occur differently. Results in this study have a potential to affect the further investigations in a broad range of plant development.

Comments for the author

The revised manuscript by Hong and Botterweg-Paredes et al. addressed most of technical comments in the first submission. Though I respect authors’ aim to obtain a more holistic picture on the interaction between REV and MAX2, in all the physiological pathways they are involved in, I have some issues which I wish authors clarify further.
This study indicates that interactions between REV and MAX2 differ in pathways being investigated, and most of these occur beyond the direct regulation of MAX2 by REV. Furthermore, their interactions seem to change depending on developmental contexts, which makes it difficult to justify the findings in one developmental process based on the findings in the other. Therefore, the scheme in Figure 7 needs either further explanation in discussion or revision. First it is unclear why gene activities of MAX2 and REV are shown in opposite direction as indicated in the top of the model. This gives an impression that one antagonizes the expression of the other. Second, the basis for color shading underlying each pathway is unclear. I do not find the reasoning or results supporting the dominance of one factor over the other in different developmental pathways. Third, the terms, ‘antagonistic’ and ‘parallel’, need further explanation, as exemplified in the following section.
For example, the model (as well as the last result section) states that REV and MAX2 play antagonistic roles in vascular patterning. But, rev10D vascular phenotype is suppressed in max2 loss-of-function mutant, meaning that MAX2 is enhancing REV function either as a downstream or

© 2020. Published by The Company of Biologists under the terms of the Creative Commons Attribution License (https://creativecommons.org/licenses/by/4.0/).

parallel component. Considering the dynamic nature of interactions between REV and MAX2, I wonder how supportive the qRT-PCR presented in the Supplemental Figure 5, as indicated in lines 445-453. The experiment was performed with 10 day old seedlings of wild type and max2-1, a stage long before the vascular patterning was examined, and the target genes measured for qRT-PCR did not seem to fit in the context of vascular patterning. Furthermore, statement in the discussion (lines 558-563 shown below) is not consistent with the statement in the results and the model: “Growth responses, such as elongation growth and branching are controlled by REV and MAX2 in an antagonistic fashion. In the senescence process, REV and MAX2 seem to act synergistically and with regard to vascular patterning it seems that MAX2 controls factors or processes that control REV activity (Fig. 6). This is supported by the finding that rev10D plants have strongly radialized vascular bundles compared to the bundles of max2 rev10D double mutant plants that resemble the wild type.”

Second revision

Author response to reviewers' comments

Reviewer 1 Advance Summary and Potential Significance to Field:
As before, this paper addresses potential interactions between two important plant developmental regulators, REV/HD-ZIPIIIs and MAX2. A range of experiments have been carried out to look at the extent to which REV and MAX2 regulated similar aspects of plant development, including gene expression analysis, shoot branching, hypocotyl elongation and senescence progression.

Reviewer 1 Comments for the Author:
The revised paper by Hong, Botterweg-Paredes et al has a number of changes to the text and figures, some of which have been highlighted by the authors. Most of the minor points originally raised have been adequately addressed and an attempt has been made to address the major points. The additional experiments and changes to the text have been partially successful in correcting the previous issues, but there are still some concerns.
Author response: We thank this reviewer for her/his insightful comments and hope that this revision satisfactorily addresses the concerns.

- In their response to the reviewers, the authors state that an interaction between REV and MAX2 may not occur via transcriptional regulation. It is, of course, true that regulatory interactions may not occur at the transcriptional level, however transcriptional level interactions are the evidence provided in this paper and used as a premise for experiments that follow in the manuscript. It is certainly possible that protein-level interactions occur, but there is no data provided to support that here. If transcriptional regulations cannot be fully relied upon and there is no protein level evidence, then it is hard to understand the rationale for the remainder of the paper. Despite this statement, however, the authors have now provided some additional evidence for the transcriptional level regulations by examining MAX2 transcripts in REV loss of function backgrounds. These provide some evidence for a fairly modest change in gene expression in certain tissues.
Author response: We are happy to read that the reviewer appreciates the data we have added to the revised version of the manuscript. In this round of revision, we have, as outlined below, focused on describing our observations and conclusions better.

The authors also provide in situ data, though I do not find the images very convincing. All images provided appear pretty similar in expression and there does not seem to be any decrease in rev5 compared to wild type. The MAX2 RNA levels in rev10d seem very similar in the leaf primordia to WT and rev5 (comparing p3 with p3, p4 with p4 etc). The only place where there is an apparent increase is in the central structure between the primordia (the meristem perhaps?) This structure is not present in the other images so it is hard to make a comparison. Is this ectopic expression of MAX2 in rev10d? With more appropriate comparative images it would be easier to evaluate this.
Author response: We have repeated the ISHs and obtained similar results. In the new images (Fig. 1D) we now show comparative images which show that *MAX2* expression is marginally lower in *rev-5* compared to Col-0. This time, no differences were observed between Col-0 and *rev10D*. We

© 2020. Published by The Company of Biologists under the terms of the Creative Commons Attribution License (https://creativecommons.org/licenses/by/4.0/).

additionally performed an analysis of the *pMAX2::GUS* reporter in the *rev10D* background which showed slightly higher GUS signal (compared to *pMAX2::GUS* in Col-0).

Overall, the new data presented partially address the original issue (that rev10d plants show more dramatic MAX2 expression changes than loss of function mutants) by identifying some circumstances in which rev/HDZIP loss of function plants sometimes exhibit reduced MAX2 expression. However, the data presented do not fully rule out the idea that many of the rev10d phenotypes may be due to ectopic expression (such as in young leaves) and may not play very significant roles in normal development.
Author response: The *rev10D* mutation renders its mRNA presumably microRNA resistant. The RNA expression domain and the domain in which REV is able to accumulate are therefore likely different. But, completely ectopic expression as in transgenic *35S::REVd* plants causes severe disturbances in development, whereas the effect of *rev10D* is rather mild. The latter fact makes it less likely that the expression changes that we have observed are entirely due to ectopic expression. Furthermore, the analysis of *pMAX2::GUS* in the *rev10D* mutant background did not show GUS signal beyond the REV expression domain (compared to *pMAX2::GUS* in Col-0).

- In conjunction with the previous point, there is some confusion in the manuscript over the nature of the proposed regulations between MAX2 and REV. Specifically, the authors state in some cases that developmental processes are independent, but also state that their data support REV regulation of MAX2 in these aspects of development. For example, lines 218-221 and lines 487-496.

Author response: We have edited respective passages to make the regulatory relationships clearer.

Some of the lack of clarity in this paper, appears to arise from conflict over whether the proposed interactions are direct or indirect. Both possibilities are potentially interesting but in different ways. Direct interactions imply a key regulatory relationship between important genes. Indirect interactions indicate that complex signaling networks in developmental processes are interconnected as parts of entire networks may overlap. With this in mind, complex interconnected networks with multiple feedback loops may explain some of the unexpected apparently antagonistic interactions. It would be helpful to establish clearly somewhere in the manuscript which developmental phenotypes likely occur as a result of direct interactions between REV and MAX2, indirect interactions, or no interaction.
Author response: We have given this aspect more thought and have carefully revised the discussion section of our manuscript. We have also added a table (Table 1) that summarizes all data.

There is a lot of good data in this paper, but it lacks a coherent interpretation. Some additional work on these aspects could be very helpful.
Author response: Thank you very much for this encouraging comment. We hope that you appreciate the changes we have introduced.

Some minor typos:
Line 83: ‘abv1’ typo
Author response: We have corrected the type.

Line 89: extra ‘as’
Author response: We have removed the extra word (in line 87).

Line 91: extra ‘is’
Author response: We have edited the sentence in line 89.

Line 100: ‘which’ should be replaced with ‘that’
Author response: We have edited the text accordingly.

Line 101: missing comma after pathway
Author response: We have inserted respective comma.

Line 103: ‘cytokinine’ typo
Author response: We have corrected the typo.

© 2020. Published by The Company of Biologists under the terms of the Creative Commons Attribution License (https://creativecommons.org/licenses/by/4.0/).

Line 204-205: ‘This contrasts the role…’ – unclear what this sentence means
Author response: We have deleted respective sentence and instead discuss the role of REV in the regulation of MAX2 in the discussion.

Line 237: extra ‘more’
Author response: We have deleted respective word.

Line 352: ‘In constistence with’ should be ‘Consistent with’
Author response: We have edited the text accordingly.

Line 437: ‘flatted’ should be ‘flattened’
Author response: We have edited the text accordingly.

Line 568: This doesn’t ‘contradict’ the REV regulation by MAX2 – it just implies that there may be feedback
Author response: We have rewritten respective sentence.

Line 692: The contributions of one of the new authors has not been described
Author response: We have update the author contributions

Reviewer 2 Advance Summary and Potential Significance to Field:
Please see my comment in 1st submission.

Reviewer 2 Comments for the Author:
In the revised version, the authors addressed some of the issues but did not provide sufficient evidence that REV indeed regulates MAX2 expression. Moreover, this shortcoming is not sufficiently presented in the manuscript: e.g. it is not mentioned in the abstract.
Author response: We wish to thank this reviewer for helping us to improve our manuscript. We have carefully assessed the abstract and have edited it to fit the manuscript. In addition, we have re-written parts of the discussion to make the regulatory relationships clearer.

The results of the extensive double mutant analyses are still very interesting to the scientific community. However, either additional experiments showing reduced MAX2 levels in rev loss- of-function mutants are needed (e.g. by further addressing the REV family redundancy issue or using the MAX2-promoter::GUS lines) or a more balanced presentation and interpretation of the data is recommended, thereby clearly outlining the limitations of the analysis.
Author response: In this new revision, we have included the MAX2::GUS reporter in the rev10D mutant background (see responses to reviewer 1). Crossing this reporter into other loss-of- function mutants is unfortunately not possible in the short timeframe. We have focussed on giving a more balanced interpretation of the data.

Reviewer 3 Advance Summary and Potential Significance to Field:
The revised manuscript by Hong and Botterweg-Paredes et al. reports comprehensive investigations of the genetic interaction between MORE AXILLARY BRANCHES 2 (MAX2) and REVOLUTA (REV), a member of Class III homeodomain leucine zipper (HD-ZIPIII) transcription factors, in various developmental processes. Though REV protein can directly bind to the promoter of MAX2, their interaction in diverse developmental processes seems to occur differently. Results in this study have a potential to affect the further investigations in a broad range of plant development.
Author response: We thank this reviewer for her/his encouraging and constructive comments.

Reviewer 3 Comments for the Author:
The revised manuscript by Hong and Botterweg-Paredes et al. addressed most of technical comments in the first submission. Though I respect authors’ aim to obtain a more holistic picture on the interaction between REV and MAX2, in all the physiological pathways they are involved in, I have some issues which I wish authors clarify further. This study indicates that interactions between REV and MAX2 differ in pathways being investigated, and most of these occur beyond the direct regulation of MAX2 by REV. Furthermore, their interactions seem to change depending on developmental contexts, which makes it difficult to justify the findings in one developmental process based on the

© 2020. Published by The Company of Biologists under the terms of the Creative Commons Attribution License (https://creativecommons.org/licenses/by/4.0/).

findings in the other. Therefore, the scheme in Figure 7 needs either further explanation in discussion or revision.
Author response: We agree with this reviewer and we have tried to come up with a unifying model that would summarize all our findings. Due to the complexity, we now decided to omit Figure 7 and instead we summarized all results in a table (Table 1). This should make it easier for the reader to get an overview of what we present in the manuscript.

First, it is unclear why gene activities of MAX2 and REV are shown in opposite direction as indicated in the top of the model. This gives an impression that one antagonizes the expression of the other.
Author response: See response to previous comment.

Second, the basis for color shading underlying each pathway is unclear. I do not find the reasoning or results supporting the dominance of one factor over the other in different developmental pathways.
Author response: See response to previous comment.

Third, the terms, 'antagonistic' and 'parallel', need further explanation, as exemplified in the following section. For example, the model (as well as the last result section) states that REV and MAX2 play antagonistic roles in vascular patterning. But, rev10D vascular phenotype is suppressed in max2 loss-of-function mutant, meaning that MAX2 is enhancing REV function either as a downstream or parallel component. Considering the dynamic nature of interactions between REV and MAX2, I wonder how supportive the qRT-PCR presented in the Supplemental Figure 5, as indicated in lines 445-453. The experiment was performed with 10 day old seedlings of wild type and max2-1, a stage long before the vascular patterning was examined, and the target genes measured for qRT-PCR did not seem to fit in the context of vascular patterning.

Author response: You are right that the qPCR-analysis used 10-day old seedlings and the vascular analysis was done in stem sections. However, the target genes are all confirmed REV direct targets and for ZPR3 it was shown that overexpression strongly affects vascular patterning, already in the early seedling stage (see Wenkel et al., Plant Cell, 2007)

Furthermore, statement in the discussion (lines 558-563 shown below) is not consistent with the statement in the results and the model: "Growth responses, such as elongation growth and branching are controlled by REV and MAX2 in an antagonistic fashion. In the senescence process, REV and MAX2 seem to act synergistically and with regard to vascular patterning it seems that MAX2 controls factors or processes that control REV activity (Fig. 6). This is supported by the finding that rev10D plants have strongly radialized vascular bundles compared to the bundles of max2 rev10D double mutant plants that resemble the wild type."
Author response: We have edited this section and adjusted the descriptions in the results section as well.

<u>Third decision letter</u>

MS ID#: DEVELOP/2019/183681

MS TITLE: Multi-level analysis of the interactions between REVOLUTA and MORE AXILLARY BRANCHES 2 in controlling plant development reveals parallel, independent and antagonistic functions

AUTHORS: Esther Botterweg, Shin-Young Hong, Jasmin Doll, Tenai Eguen, Anko Blaakmeer, Sanne Matton, Yakun Xie, Bjoerg Skjoeth Lunding, Ulrike Zentgraf, Chunmei Guan, Yuling Jiao, and Stephan Wenkel
ARTICLE TYPE: Research Article

I am happy to tell you that your manuscript has been accepted for publication in Development, pending our standard ethics checks.

© 2020. Published by The Company of Biologists under the terms of the Creative Commons Attribution License (https://creativecommons.org/licenses/by/4.0/).

Reviewer 2

Advance summary and potential significance to field

See previous reviews.

Comments for the author

The authors´ conclusions on their data are phrased more carefully now, placing more emphasis on the only very mild change in MAX2 expression in the rev loss-of-function mutant. Though the data, therefore, remain descriptive and cannot support a holistic model on an interaction between MAX2 and REV, the double mutant analysis does provide interesting phenotypic data.

Minor comment:
The text in the abstract contains word-order problems in lines 42 and 44. Please correct.

Reviewer 3

Advance summary and potential significance to field

As indicated in the previous review, this study could benefit the research community by providing comprehensive information about the genetic interaction between MAX2 and REV in many different developmental processes.

Comments for the author

In this manuscript, the genetic interaction between MAX2 and REV was analyzed based on phenotypic analyses in multiple developmental processes. Phenotype and a type of regulatory relationship in each process are summarized in Table 1. It shows that their interaction is quite complicated, which cannot be explained by the molecular regulation presented at the beginning part of results. I think dropping the model in this revision was appropriate considering it had a potential to mislead the data interpretation. Nevertheless, such incoherence in molecular regulation and genetic interaction makes the manuscript lack a whole unity. Authors addressed the comments twice already, thus I do not have any further specific comment.

© 2020. Published by The Company of Biologists under the terms of the Creative Commons Attribution License (https://creativecommons.org/licenses/by/4.0/).
